# Fatty links between multisystem proteinopathy and small VCP-interacting protein

**DOI:** 10.1038/s41420-024-02118-9

**Published:** 2024-08-08

**Authors:** Firyal Ramzan, Ashish Kumar, Fatima Abrar, Rachel A. V. Gray, Zurie E. Campbell, Lucia Meng Qi Liao, Anthony Dang, Oluwadurotimi Akanni, Colm Guyn, Dale D. O. Martin

**Affiliations:** Department of Biology, University of Waterloo, Waterloo, ON Canada

**Keywords:** Neurological disorders, Cell death, Lipids

## Abstract

Multisystem proteinopathy (MSP) is a rare, dominantly inherited disorder that includes a cluster of diseases, including frontotemporal dementia, inclusion body myopathy, and Paget’s disease of bone. MSP is caused by mutations in the gene encoding valosin-containing protein (VCP). Patients with the same mutation, even within the same family, can present with a different combination of any or all of the above diseases, along with amyotrophic lateral sclerosis (ALS). The pleiotropic effects may be linked to the greater than 50 VCP co-factors that direct VCP’s many roles in the cell. Small VCP-interacting protein (SVIP) is a small protein that directs VCP to autophagosomes and lysosomes. We found that SVIP directs VCP localization to lysosomes in an acylation-dependent manner. We demonstrate that SVIP is myristoylated at Glycine 2 and palmitoylated at Cysteines 4 and 7. Acylation of SVIP is required to mediate cell death in the presence of the MSP-associated VCP variant (R155H-VCP), whereas blocking SVIP myristoylation prevents cytotoxicity. Therefore, SVIP acylation may present a novel target in MSP.

## Introduction

Point mutations in the gene encoding for valosin-containing protein (VCP) have been causally and correlatively linked to numerous autosomal dominant neurodegenerative diseases. These include VCP diseases and multisystem proteinopathy (MSP), in which patients may present with one or any combination of inclusion body myopathy, Paget’s disease of bone, and frontotemporal dementia, while some patients will also develop amyotrophic lateral sclerosis (ALS) [1], Parkinson’s disease [2], and other diseases [3].

VCP is an AAA+ (ATPases Associated with diverse cellular Activities) ATPase that exists and functions as a homohexamer. VCP is highly coordinated by its many co-factors, which can dynamically deploy VCP to different organelle systems, as necessary, for cell maintenance and homeostasis, including ubiquitin-dependent signaling and ER-associated degradation (ERAD), macroautophagy, and selective autophagy (mitophagy and lysophagy) [4]. Autophagy is a tightly controlled process that delivers damaged organelles and toxic proteins to the lysosome for degradation through the formation of double-membraned vesicles called autophagosomes [5, 6]. Sequestration of proteins from the cytosol into membrane-enclosed vesicles (autophagosomes) is critical for proteostasis and cellular health. VCP has been implicated in cargo loading and autophagolysosome fusion [4, 7, 8]. During ERAD, a quality control mechanism that involves the retrotranslocation of misfolded proteins across the ER membrane, VCP mediates the extraction of proteins from the ER [9, 10]. In MSP, VCP’s regulation of the maturation of ubiquitin-containing autophagosomes in autophagy is impaired [11]. VCP’s functions in autophagy are regulated by its many co-factors [12], such as YOD1 and UBXD1, which mediate VCP’s function in autophagic clearance of lysosomes [13].

The VCP co-factor small VCP-interacting protein (SVIP) regulates VCP’s function in the activation of autophagy and negatively regulates ERAD [14–17]. SVIP is a small protein that binds the N-terminal domain of VCP [18, 19], and can redirect VCP localization to autophagosomes [17]. Recent studies in *Drosophila melanogaster* (*D. melanogaster*) showed that reducing SVIP disrupts muscle lysosomal function, and causes muscular and neuromuscular degeneration, motoneuronal degeneration, motor dysfunction, and reduced lifespan [16]. These studies further characterized an SVIP mutation identified in patients with sporadic frontotemporal dementia and confirmed its pathogenicity in *D. melanogaster* [16].

Previous studies have linked SVIP localization and function to predicted N-myristoylation [14, 19], the non-reversible addition of the 14-carbon saturated fatty acid myristate to N-terminal glycines by *N*-myristoyl transferase (NMT) enzymes 1 and 2 (NMT1 and NMT2) [20, 21]. N-myristoylation, herein referred to as myristoylation, can occur either co-translationally, after the removal of the initiator methionine on the nascent polypeptide, or post-translationally, following the exposure of an N-terminal glycine of the C-terminal product of proteolyzed proteins [21]. The myristate moiety promotes weak membrane binding and often requires a second membrane binding site for stable membrane attachment. Therefore, myristoylated proteins typically undergo additional forms of lipidation at downstream sites. In the case of SVIP, two cysteines immediately downstream of the glycine are predicted to undergo S-acylation (CSS-Palm 3.0 [22]). Similar to myristoylation, S-acylation involves the addition of saturated fatty acids, but via a thioester bond on cysteine residues, making it labile and reversible [6, 23]. The 16-carbon fatty acid palmitate is typically used; therefore, this modification is more commonly referred to as S-palmitoylation or simply palmitoylation.

Although there is no known consensus sequence for S-acylation, N- and C-terminal cysteines are often S-acylated, especially when adjacent to a myristoylated site [23, 24]. In contrast, myristoylation typically occurs on N-terminal glycines found within a G*XXX*T/S/C consensus motif, where *X* can be almost any amino acid. Prolines and large bulky amino acids are not usually tolerated in these positions. Therefore, human SVIP does not have an ideal myristoylation consensus sequence (MGLCFPCPG). Regardless, SVIP is still predicted to be myristoylated (TermiNator3 [25, 26], SVMyr [27]). Previous reports that the N-terminus is required for SVIP localization suggested SVIP is fatty acylated [14, 19]. However, no studies have directly detected either myristoylation or S-acylation of SVIP. Studies have indirectly investigated the role of myristoylation on SVIP function as a VCP co-factor, such that modifying the N-terminus affects VCP localization, which could involve a number of N-terminal modifications [17]. We hypothesized that SVIP is S-acylated at Cysteines 4 and 7, and myristoylated at Glycine 2.

In this study, we confirm that SVIP is both myristoylated and S-acylated. Moreover, we find that localization of both SVIP and VCP is modulated by SVIP acylation. Further, SVIP has an acylation-dependent cytotoxic interaction with R155H-VCP, a disease-associated variant of VCP.

## Results

### SVIP is both myristoylated and palmitoylated

**Fig. 1:**
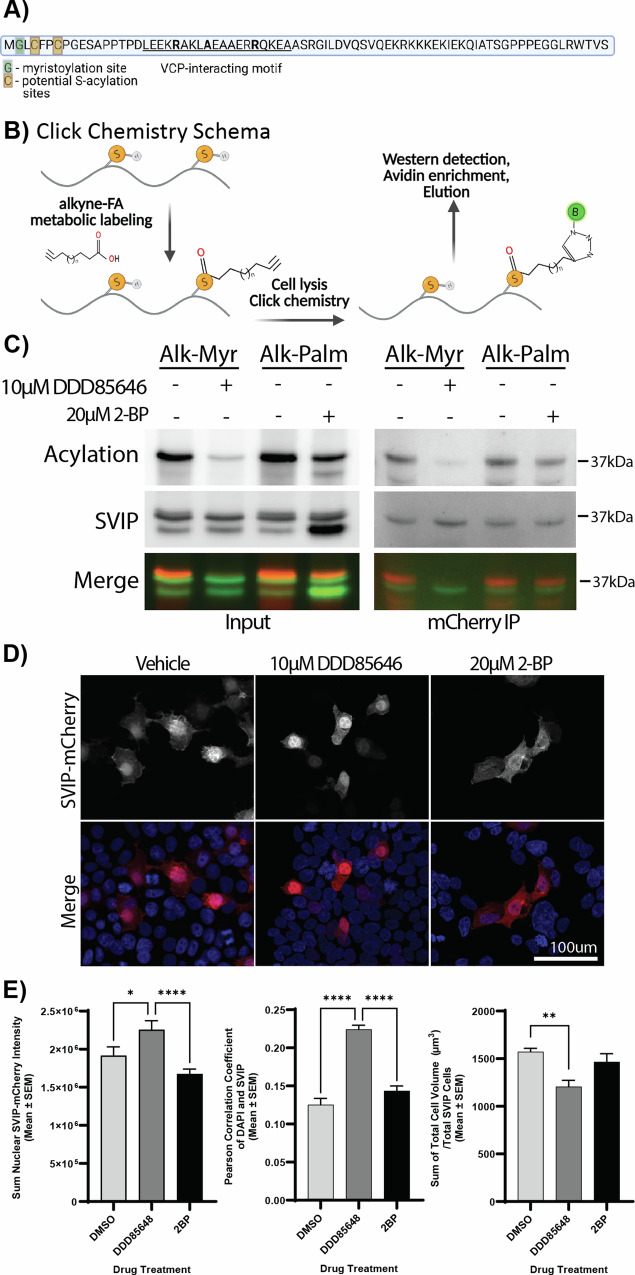
SVIP is myristoylated and palmitoylated. **A** Amino acid sequence of SVIP. SVIP is a small 77 amino acid long protein that is predicted to undergo myristoylation at Glycine 2 (G; Green; TermiNator and SVMyr) and palmitoylation at Cysteines 4 and 7 (C; orange; CSS-Palm 3.0). The VCP-interacting motif (VIM), composed of amino acids 18–35, is underlined and essential amino acids are bolded. **B** Depiction of the click chemistry method used to detect SVIP palmitoylation and myristoylation; FA fatty acid. Prepared using Biorender.com. **C** SVIP-mCherry incorporation of alkyne-myristate and alkyne-palmitate was detected by click chemistry and is sensitive to their respective myristoylation (DDD85646) and palmitoylation (2-BP) inhibitors in HEK293T cells. **D** Myristoylation or palmitoylation inhibition alters SVIP-mCherry localization in HEK293T cells. **E** Quantification of (left) nuclear mCherry intensity, (middle) Pearson correlation coefficient between SVIP and DAPI, and (right) Total cell volume/total SVIP cells, on an average of 50 SVIP cells per condition from three biological repeats. The significance bars represent Tukey’s post-hoc analyses (*p < 0.05, **p < 0.01, ****p  < 0.0001).

SVIP has three predicted acylation sites, G2, C4, and C7 (Fig. 1A). Using click chemistry (Fig. 1B), we confirmed that SVIP-mCherry is both myristoylated and palmitoylated (Fig. 1C). The acylation signal was also prevented by pretreatment with acylation inhibitors 15 min before alkyne-fatty acid labeling. Specifically, treatment with the myristoylation inhibitor DDD85646 reduced SVIP myristoylation with the alkyne-myristate label, while treatment with the non-specific palmitoylation inhibitor 2-bromopalmitate (2-BP) reduced SVIP palmitoylation with the alkyne-palmitate label (Fig. 1C). Incorporation of the palmitate analog confirms that SVIP undergoes palmitoylation.

When expressed alone, SVIP-mCherry appears to localize to membranes throughout the cell (Fig. 1C). However, upon treatment with DDD85646, SVIP-mCherry becomes diffusely expressed throughout the cell. Like other short acylated peptides appended to a fluorescent protein [28–31], blocking myristoylation results in SVIP-mCherry significantly relocalizing to the nucleus (an ANOVA comparing total nuclear intensity across the three conditions was significant (F (2, 1252) = 9.772, *p* < 0.0001), with Tukey’s post-hoc analysis revealing the DDD85646-treated sample having a higher nuclear intensity than DMSO (*p* < 0.01) and 2-BP (*p* < 0.001) treated samples), similar to mCherry alone. These data are further recapitulated by colocalization between SVIP-mCherry and DAPI. Specifically, the Pearson correlation coefficient (PCC) between SVIP-mCherry and DAPI shows a significant difference by ANOVA (F (2, 1492) = 69.58, *p* < 0.0001), with Tukey’s revealing a higher PCC in the DDD85646-treated sample compared to DMSO (*p* < 0.0001) and 2-BP (*p* < 0.0001) treatments. Blocking SVIP-mCherry palmitoylation with 2-BP only partially altered SVIP-mCherry localization (Fig. 1D). Interestingly, DDD85646 treatment significantly reduced average total cell volume over total SVIP cells in comparison to DMSO treatment (Tukey’s, *p* < 0.01; Fig. 1E). This suggests myristoylation is required for palmitoylation of SVIP-mCherry, and blocking myristoylation with DDD85646 blocks nearly all SVIP-mCherry acylation (both palmitoylation and myristoylation) while blocking palmitoylation with 2-BP only blocks palmitoylation, allowing SVIP-mCherry to partially localize to membranes. Thus, SVIP-mCherry follows the standard pattern of fatty acylation at the N-terminus [28–31].

### SVIP is myristoylated at Glycine 2 and palmitoylated at Cysteines 4 and 7

**Fig. 2:**
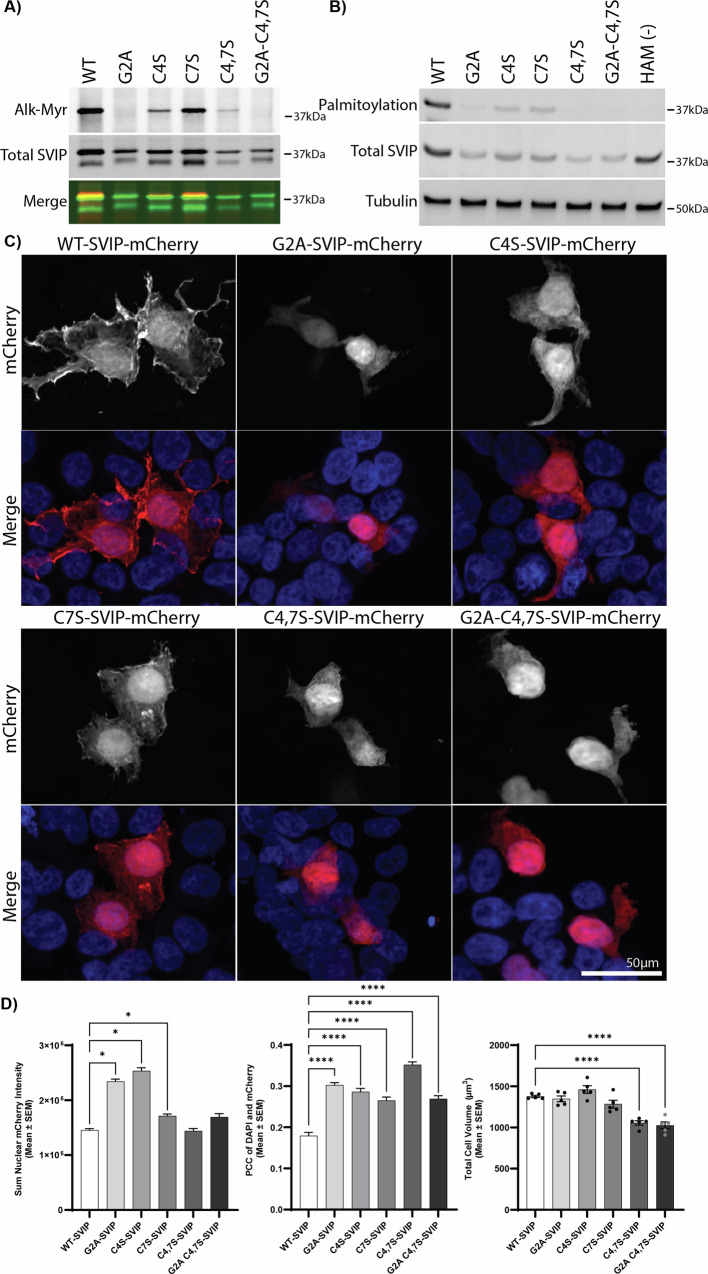
SVIP is myristoylated at Glycine 2 and palmitoylated at Cysteines 4 and 7. Point mutations at the indicated positions were made to block myristoylation (G2) and palmitoylation (C4,7S) in SVIP-mCherry. HEK293T cells expressing these mutations were used to detect **A** myristoylation by click chemistry using alkyne-myristate and **B** palmitoylation by ABE. **C** SVIP-mCherry localization was acquired by fixed cell confocal microscopy. **D** Quantification of (left) nuclear mCherry intensity, (middle) PCC of DAPI and SVIP, and (right) total cell volume, on an average of 288 SVIP cells per condition from two biological repeats. The significance bars represent Tukey’s post-hoc analyses (*p < 0.05, ****p < 0.0001).

To confirm that myristoylation is required for palmitoylation and to verify the sites of acylation, we introduced an alanine mutation at G2 (G2A) and serine mutations at C4 and C7 (C4S and C7S) and detected myristoylation by click chemistry (Fig. 2A) and palmitoylation (by acyl-biotin exchange; ABE) (Fig. 2B). Serine was used because it is more sterically similar to cysteine than alanine, and less likely to interfere with the myristoylation consensus sequence [28]. We confirmed that SVIP-mCherry myristoylation requires the essential glycine at position 2 (G2A and G2A-C4,7S) using alkyne-myristate labeling and click chemistry. While the C7S substitution did not affect myristoylation, the C4S and C4,C7S mutations consistently had a lower myristoylation signal than WT (Fig. 2A). This suggests that the cysteine in position 4 is likely required for recognition by NMT. To ensure that the substitution to serine was not the cause, we also generated a cysteine to alanine mutation that also reduced myristoylation (data not shown), indicating that cysteine at position 4 is required for myristoylation of SVIP-mCherry.

An ABE assay was performed to assess palmitoylation more directly. As predicted, point mutations at C4 and C7 reduced SVIP palmitoylation, but not completely, whereas the double mutation (C4,7S) completely blocked palmitoylation, indicating SVIP-mCherry is palmitoylated at C4 and C7. Palmitoylation was nearly entirely inhibited when myristoylation was blocked in G2A (Fig. 2B), confirming that myristoylation is required for downstream palmitoylation. The faint signal that remained is likely due to partial palmitoylation at the most distal C7 site. This confirms that myristoylation is required for SVIP palmitoylation.

We next determined whether SVIP’s acylation regulates its localization in HEK293T cells. WT-SVIP localized heavily on membrane ruffles and throughout the cell, whereas non-acylated SVIP (G2A) was both nuclear and diffuse within the cell (Fig. 2C). Substitution at the C4 site had a similar effect as G2A, likely due to reduced myristoylation and palmitoylation. C7S-SVIP had an intermediate effect with some membrane-specific localization, but not as prominent as WT-SVIP. The combined acylation mutants, C4,7S and G2A-C4,7S, had a general diffuse pattern of localization, likely due to the presence of either one or both of the G2A and C4S mutations (Fig. 2C), which block myristoylation and palmitoylation. This effect was significant (Fig. 2D), as the total nuclear intensity (F (5, 12525) = 105.3, *p* < 0.0001) in the G2A-SVIP (Tukey’s, *p* < 0.05) and C4S-SVIP (Tukey’s, *p* < 0.05) mutants was higher in comparison to WT-SVIP-mCherry. This was also the case with C7S-SVIP-mCherry (Tukey’s, *p* < 0.05), where the intermediate appearance of change in localization was significant in comparison to WT-SVIP. The multi-point acylation mutants (C4,7S and G2A-C4,7S) were both comparable to WT-SVIP-mCherry. Additionally, in comparison to WT-SVIP-mCherry, the colocalization of SVIP-mCherry and DAPI (F (5, 3417) = 59.02, *p* < 0.0001) was higher with all SVIP acylation mutants, suggesting that the prevention of either or both palmitoylation and myristoylation increased nuclear localization of all mutants. Conversely, the total cell volume (F (5, 24) = 23.19, *p* < 0.0001) of the multi-point mutants C4,7S-SVIP-mCherry (Tukey’s, *p* < 0.00001) and G2A-C4,7S-SVIP-mCherry (Tukey’s *p* < 0.0001) was significantly lower than WT-SVIP-mCherry. In contrast, the single acylation mutants were comparable to WT-SVIP-mCherry. This suggests the overall cell size may be smaller with multiple acylation mutations compared to WT-SVIP-mCherry. This reduced cell size may indicate a potential change in function through blocking fatty acylation of SVIP.

### SVIP-mCherry directs VCP-GFP localization and is SVIP acylation dependent

**Fig. 3:**
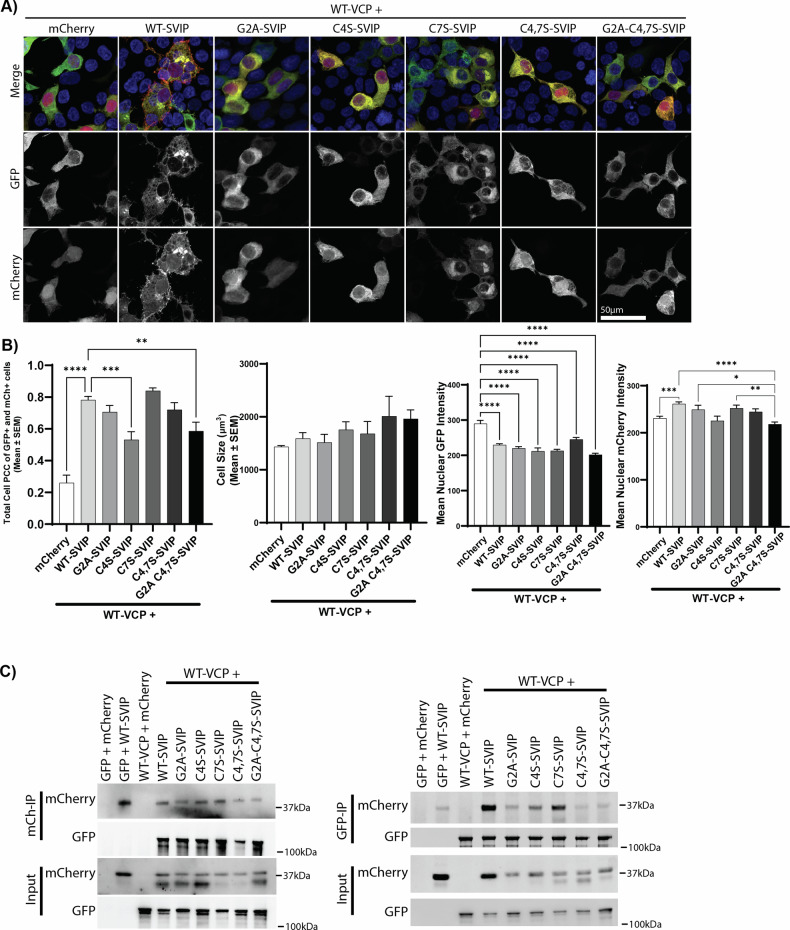
SVIP acylation is required to direct VCP localization. **A** HEK293T cells co-expressing WT-VCP-GFP and SVIP-mCherry acylation mutants were imaged by confocal microscopy. **B** Quantification of (left) Pearson correlation coefficient of WT-VCP and mCherry. The significance bars represent Tukey’s post-hoc analyses (*p < 0.05, **p < 0.01, ***p < 0.001, ****p < 0.0001). The comparison between WT-VCP + mCherry and the rest of the conditions is not shown for simplicity; however, all conditions were significantly increased at *p* < 0.001, (right) quantification of cell size measurements on an average of 152 total SVIP and VCP cells for each condition from two biological repeats. **C** Co-immunoprecipitation with anti-mCherry (left) and anti-GFP (right) from cell lysates of HEK293T cells expressing the combinations of WT-VCP-GFP with SVIP-mCherry acylation mutants.

To assess how acylation may regulate SVIP colocalization with VCP, SVIP-mCherry and VCP-GFP were co-expressed in HEK293T cells (Fig. 3A). Alone, WT-VCP-GFP was diffusely expressed throughout the cell (Fig. 3A). When expressed with WT-SVIP-mCherry, both VCP-GFP and SVIP-mCherry localize to vesicles reminiscent of autophagosomes (Supplementary Videos 1–3), as previously described [17]. Strikingly, the formation of these vesicles was dependent on SVIP-mCherry acylation. No vesicles were detected in the presence of G2A-SVIP-mCherry (Supplementary Videos 4–6). However, regardless of the acylation mutant, VCP-GFP and SVIP-mCherry localization overlapped, suggesting the proteins still interact (Fig. 3B, left), without modifying total cell size (Fig. 3B, right). Specifically, colocalization between WT-VCP-GFP and all acylated forms of SVIP-mCherry significantly increased compared to WT-VCP-GFP and mCherry alone (Fig. 3B, left; *p* < 0.001 not shown in the figure, ANOVA, F (6, 95) = 22.47, *p* < 0.0001) of the PCC. Interestingly, the most significant reduction in colocalization between VCP-GFP and SVIP-mCherry was observed in C4S-SVIP-mCherry (*p* < 0.001) and G2A-C4,7S-SVIP-mCherry (*p* < 0.01), suggesting that the C4S mutation reduces VCP and SVIP overlap and, possibly, interaction.

To confirm the interaction, we performed co-immunoprecipitation studies using WT-VCP-GFP and the acylation mutants of SVIP-mCherry (Fig. 3C). When immunoprecipitating SVIP-mCherry, the acylation status did not appear to affect the co-immunoprecipitation of VCP-GFP (Fig. 3C, left). In contrast, when VCP-GFP was immunoprecipitated, SVIP-mCherry mutants that had reduced or no myristoylation (G2A, C4,7S, and G2A-C4,7S), and, therefore, little to no palmitoylation, were reduced to background levels (Fig. 3B, right). This suggests that VCP is a primary interactor of SVIP, and the interaction is independent of acylation. However, because VCP has many interactors, non-acylated SVIP may not have the opportunity to interact with VCP and, therefore, less SVIP co-immunoprecipitates with VCP.

### SVIP-mCherry and R155H-VCP-GFP have an acylation-dependent cytotoxic interaction

**Fig. 4:**
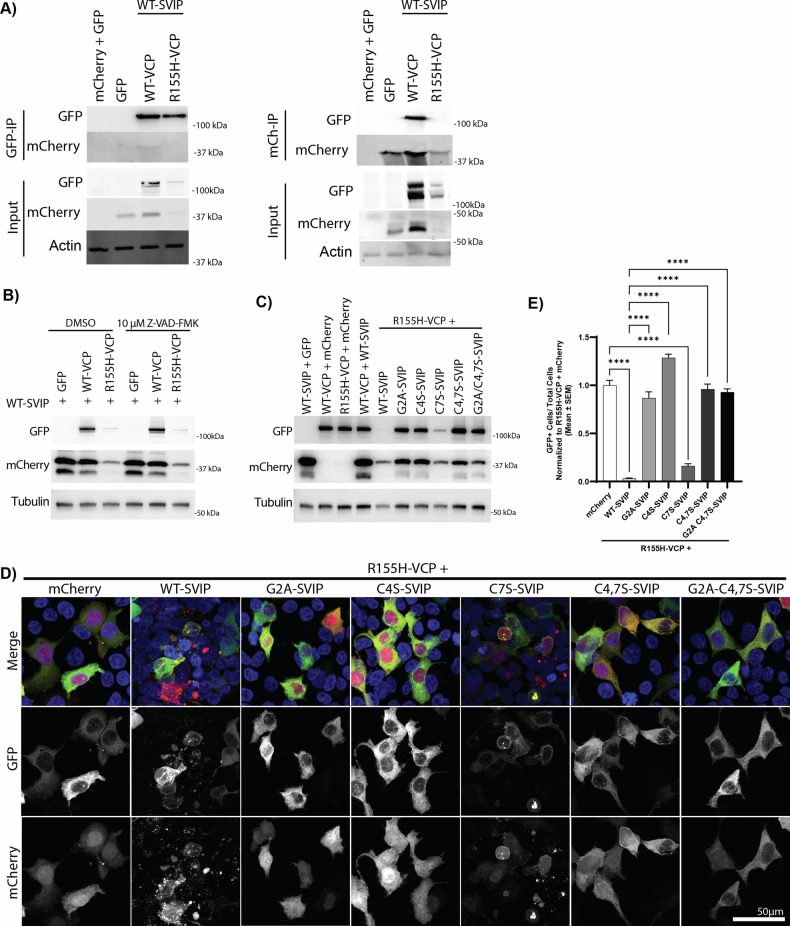
The toxic interaction of SVIP and R155H-VCP is rescued by blocking SVIP acylation. **A** Coexpression of SVIP-mCherry with mutant VCP-GFP (R155H or A232E) was toxic to HEK293T cells and led to the degradation of the two proteins. **B** Toxicity was not rescued by caspase inhibition with the general caspase inhibitor Z-VAD-FMK. The toxic effects were reversed when acylation was blocked and visualized **C** biochemically or **D** by confocal microscopy. **E** Quantification of VCP+ cells/total cells, normalized to R155H-VCP + mCherry, on cells from three biological repeats. The significance bars represent Tukey’s post-hoc analyses (****p < 0.0001).

Next, we sought to determine if the acylation-dependent effect of SVIP and VCP was maintained in VCP disease. The R155H-VCP variant accounts for almost 50% of VCP disease cases [11]. Surprisingly, when R155H-VCP-GFP was co-expressed with WT-SVIP-mCherry, we detected rapid cell deterioration, ultimately leading to cell death soon after the expression of both proteins (Supplementary Video 7). These findings were replicated biochemically (Fig. 4A). Specifically, protein levels of both R155H-VCP-GFP and WT-SVIP-mCherry were substantially reduced when co-expressed. Furthermore, this rapid cell death was resistant to caspase inhibition (Fig. 4B), suggesting caspase-independent cell death. Attempts to block cell death using lysosome (bafilomycin A1 and pepstatin A) or proteasome inhibitors (MG132), did not restore protein levels (data not shown). To determine if this cytotoxicity was acylation dependent, we co-expressed R155H-VCP-GFP with acylation mutants of SVIP-mCherry. Notably, blocking SVIP acylation (G2A, C4S, C4,7S, and G2A-C4,7S) rescued the cytotoxic effect as measured through protein levels biochemically (Fig. 4C) and through GFP-positive cell counts (Fig. 4D), with the exception of C7S-SVIP-mCherry, which appeared to have an intermediate effect.

Due to the rapid caspase-independent cell death, cell viability was quantified using measurements of VCP-GFP-positive cells (Fig. 4E), since cells died almost immediately after VCP-GFP expression. VCP-GFP-positive cells were significantly reduced when R155H-VCP was co-expressed with WT-SVIP (Tukey’s, *p* < 0.0001) and with C7S-SVIP (Tukey’s, *p* < 0,0001) in comparison to the R155H-VCP with mCherry alone. However, while C7S-SVIP resulted in a visually intermediate feature, statistically, it was not different from WT-SVIP. On the other hand, VCP-GFP-positive cells were significantly increased when R155H-VCP was co-expressed with G2A-SVIP (Tukey’s, *p* < 0.0001), C4S-SVIP (Tukey’s, *p* < 0.0001), C4,7S-SVIP (Tukey’s, *p* < 0.0001), and G2A-C4,7S-SVIP (Tukey’s, *p* < 0.0001) in comparison to WT-SVIP. This indicates that the cell death caused by R155H-VCP-GFP with WT-SVIP is acylation dependent.

### Treatment with acylation inhibitors rescues cell death

**Fig. 5:**
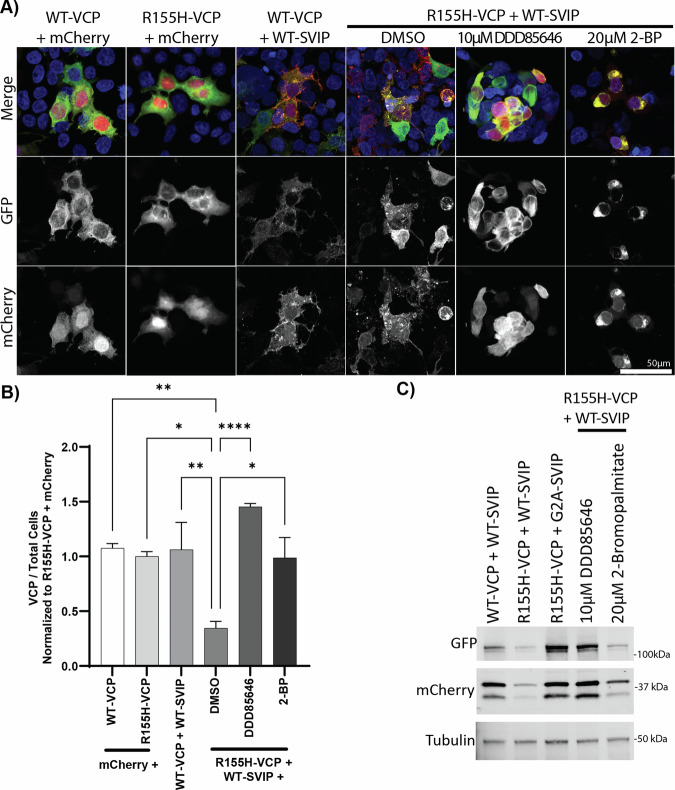
Global inhibition of palmitoylation or myristoylation rescues toxicity. HEK293T cells co-transfected with R155H-VCP-GFP and WT-SVIP-mCherry were treated with vehicle (DMSO), 10 µM DDD85646, and 20 µM 2-BP overnight for 18 h. **A** Cells treated with inhibitors were imaged by fixed cell confocal microscopy. **B** Quantification of VCP+ cells/total cells, normalized to R155H-VCP + mCherry, on cells from two biological repeats. The significance bars represent Tukey’s post-hoc analyses (*p < 0.05, **p < 0.01, ****p < 0.0001). **C** Western blot results depict the rescue in protein expression levels when treated with DDD85646 and moderate rescue with 2-BP treatment.

Because the C4S substitution in SVIP also decreased myristoylation (Fig. 2A), it was difficult to determine if the protective effect of blocking acylation was mediated by palmitoylation or myristoylation. This was compounded by the fact that myristoylation is required for downstream palmitoylation at C4 and C7 (Fig. 2B). To determine if palmitoylation was necessary, cells co-expressing R155H-VCP-GFP and WT-SVIP-mCherry were treated with 10 µM DDD85646 or 20 µM 2-BP to inhibit myristoylation or palmitoylation, respectively. Treatment with the myristoylation inhibitor (DDD85646) completely prevented cell death (Fig. 5). In contrast, treatment with the palmitoylation inhibitor (2-BP) appeared to have an intermediate effect on the toxic interaction such that, upon fixation, cells were found at the intermediate stage of rounding up (Fig. 5A). Quantifying VCP-GFP-positive cells over total cells normalized to the R155H-VCP-GFP with mCherry condition further reinforced these findings. Specifically, VCP-GFP-positive cells were significantly reduced when WT-SVIP was co-expressed with R155H-VCP-GFP in comparison to the WT-VCP-GFP with WT-SVIP-mCherry condition (*p* < 0.01, following a significant ANOVA, F (5, 24) = 7.361, *p* < 0.001, Tukey’s post-hoc), and this reduction was rescued in the presence of DDD85646 (*p* < 0.0001) or 2-BP (*p* < 0.05). These findings were also recapitulated biochemically (Fig. 5B). Specifically, protein levels of both R155H-VCP-GFP and WT-SVIP-mCherry were high when myristoylation was inhibited (G2A-SVIP, DDD85646 treatment), and substantially reduced when treated with vehicle (DMSO) or the palmitoylation inhibitor (2-BP) (Fig. 5B). This suggests that the cell death observed upon coexpression of R155H-VCP-GFP and WT-SVIP-mCherry is largely dependent on myristoylation, while inhibiting palmitoylation provides some protection.

### Blocking the SVIP VCP-interaction motif (VIM) rescues cell death

**Fig. 6:**
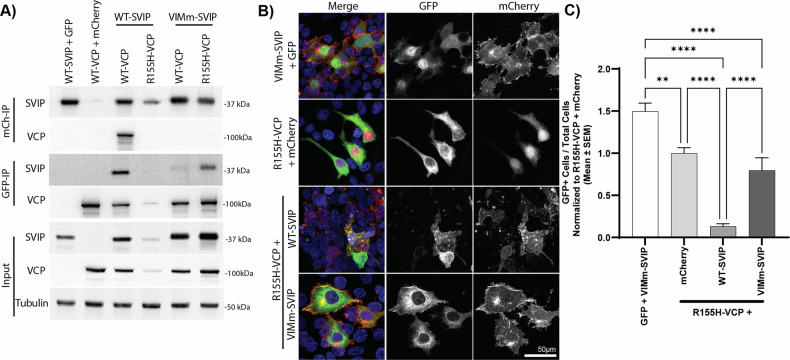
Blocking SVIP’s interaction with VCP rescues toxicity. SVIP-mCherry with multiple site mutations in the VCP-interacting motif (VIMm-SVIP-mCherry) was generated and co-expressed with R155H-VCP-GFP in HEK293T cells. **A** Co-immunoprecipitation results depict that the VIMm-SVIP-mCherry does not interact with WT-VCP, but does with R155H-VCP and rescues R155H-VCP protein expression. **B** Fixed cells were imaged using the Nikon AXR confocal microscope at 20× magnification. **C** Quantification of VCP+ cells/total cells, normalized to R155H-VCP + mCherry, on cells from three biological repeats. The significance bars represent Tukey’s post-hoc analyses (**p < 0.01, ****p < 0.0001).

To determine if the cytotoxic effect was dependent on the interaction of VCP and SVIP, three point mutations were made to the VIM region (R22E, A26V, R32E ^18^LEEK**R**AKL**A**EAAER**R**QKE^35^; Fig. 1A) in SVIP-mCherry (VIMm-SVIP-mCherry). As previously published [14], this caused VIMm-SVIP-mCherry to migrate slightly higher by SDS–PAGE and was sufficient to prevent WT-VCP-GFP from co-immunoprecipitating with VIMm-SVIP-mCherry (Fig. 6A). However, VIMm-SVIP-mCherry had a reduced but still prominent interaction with R155H-VCP-GFP that was only detected in the GFP immunoprecipitate but not the mCherry immunoprecipitate (Fig. 6A). This reduction was sufficient to rescue cell death indicated by microscopy (Fig. 6B, C) and rescue the VCP and SVIP proteins biochemically (Fig. 6A). By microscopy, VIMm-SVIP-mCherry still localized to intracellular membranes (Fig. 6B, top row), reminiscent of WT-SVIP-mCherry (Figs. 1–5) and did not overlap with R155H-VCP-GFP, which was dispersed throughout the cell (Fig. 6B, bottom row), similar to when it is expressed on its own (Fig. 6B, second row). This data is further supported by quantification of the ratio of VCP-positive cells over total cells, normalized to R155H-VCP + mCherry condition (Fig. 6C). The quantification confirmed a reduction of VCP-positive cells when R155H-VCP was co-expressed with WT-SVIP in comparison to coexpression with mCherry (F (3, 56) = 34.36, *p* < 0.0001; Tukey’s *p* < 0.0001). Further, VCP-positive cells were significantly recovered when R155H-VCP was co-expressed with VIMm-SVIP compared to coexpression with WT-SVIP (Tukey’s, *p* < 0.0001). There was also a significant reduction in GFP signal when R155H-VCP was co-expressed with mCherry in comparison to GFP co-expressed with VIMm-SVIP (*p* < 0.01); however, this is likely due to the higher expression of the empty GFP vector.

### Lysosome localization & cytotoxicity is dependent on SVIP acylation

**Fig. 7:**
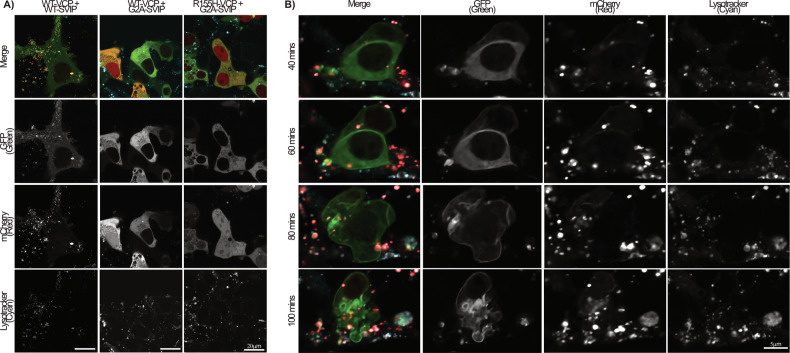
Lysosome targeting and toxicity is acylation dependent. HEK293T expressing WT or G2A-SVIP-mCherry with WT or R155H-VCP-GFP were incubated with LysoTracker Deep Red (cyan) soon after transfection and imaged **A** the next day or **B** overnight using the Nikon AXR confocal microscope at 60× magnification.

Using live-cell microscopy (Fig. 7, Supplementary Videos 8–11), we observed that acylation of WT-SVIP was required to localize SVIP-mCherry and WT-VCP-GFP to LysoTracker stained lysosomes (Fig. 7A, Supplementary Video 8), similar to previously published data demonstrating SVIP requires its N-terminus/G2 site to relocalize VCP to plasma membranes, membrane-bound vesicles, and cell vacuoles [17]. When SVIP acylation was blocked (G2A-SVIP-mCherry), the two proteins no longer localized to the lysosome and appeared diffuse within the cytoplasm, suggesting SVIP is required to target the two proteins to the lysosome (Fig. 7A, Supplementary Videos 9 and 10). However, when WT-SVIP-mCherry was co-expressed with R155H-VCP-GFP (Fig. 7B, Supplementary Video 11), the cells died soon after VCP expression was detected (~60–100 min). Initially, WT-SVIP-mCherry could be seen localizing to the lysosomes in cells not expressing R155H-VCP-GFP. Once GFP was detected, the cells quickly began dying. No apparent mutant VCP was detected at the lysosome with WT-SVIP-mCherry. Again, this cytotoxicity was reversed after blocking SVIP acylation and localization to the lysosome. This suggests that SVIP must localize to the lysosome to mediate cytotoxicity in the presence of mutant VCP.

### VCP and SVIP localization are replicated in neurons

**Fig. 8:**
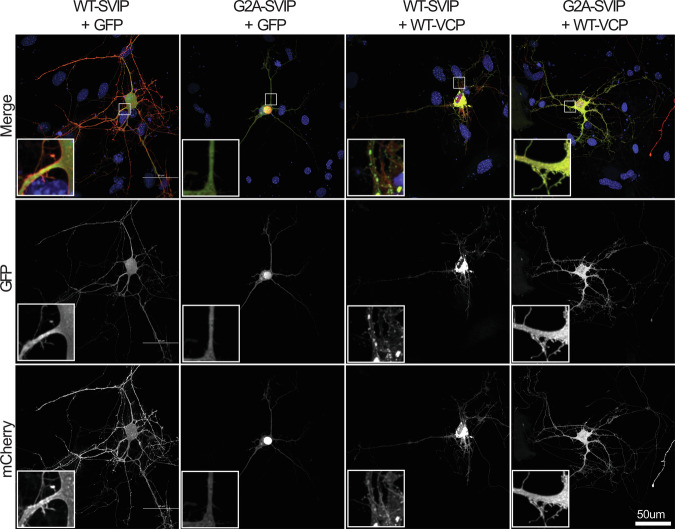
SVIP-mCherry and VCP-GFP expression in hippocampal neurons. Primary hippocampal neurons derived from FVB mice were transfected with WT- or G2A-SVIP-mCherry and GFP or WT-VCP-GFP. Neurons were imaged by fixed cell confocal microscopy at 60×. Insets depict a 50% zoomed-in view of neurites at the base of the cell body.

Mouse-derived primary hippocampal neurons were transfected with SVIP-mCherry and WT-VCP-GFP (Fig. 8). As seen in HEK293T cells, WT-SVIP-mCherry was found along membranes, particularly along the axons and dendrites, while G2A-SVIP-mCherry was nuclear and dispersed diffusely throughout the cell body, axons, and dendrites. Again, coexpression of WT-VCP-GFP with WT-SVIP-mCherry resulted in autophagosome-like vesicles found in the cell body and throughout the neurites (Fig. 8, column 3 inset), which are no longer detected with coexpression of G2A-SVIP-mCherry and WT-VCP-GFP. Blocking SVIP-mCherry acylation resulted in a general diffuse expression pattern of both proteins.

## Discussion

Previous studies have indicated that the N-terminus of SVIP is required for SVIP localization and affects VCP localization [17]. Here we confirm that human SVIP is myristoylated at G2, which is required for the downstream palmitoylation at sites C4 and, to a lesser degree, C7 (Fig. 2). In turn, SVIP acylation is required for SVIP localization and can regulate VCP localization when co-expressed. Together, as previously indicated [17], SVIP-mCherry and VCP-GFP localize to autophagosome-like vesicles and is dependent on SVIP-mCherry acylation (Fig. 3, Supplementary Videos 1–6). Furthermore, the combination of SVIP with disease variants of VCP is lethal to the cell through a caspase-independent form of cell death that is SVIP acylation dependent.

SVIP’s cellular localization is differentially regulated by myristoylation and palmitoylation. Blocking myristoylation using DDD85646 treatment relocalized SVIP to the nucleus (Fig. 1D, E). While the DAPI-SVIP PCC significantly increased with DDD85646 treatment, the overall PCC value is relatively low, at values between 0.1 and 0.25. This may be explained by the measurement methods used. Specifically, a mask around DAPI was used to measure the colocalization of mCherry within DAPI-stained areas. This was done to overcome the exaggerated concentration of mCherry detected by the program due to subtle differences in cell size observed with DDD85646 treatment in whole-cell measurements. Further, while SVIP-mCherry shows increased localization to the nucleus with DDD85646 treatment, it was dispersed throughout the nucleus with heavy localization at the nuclear membranes. Additionally, DAPI binds DNA and abstains from staining low-DNA areas of the nucleus (e.g., nucleoli), resulting in “black holes” in the nucleus. These regions, however, do overlap with SVIP-mCherry signal. These scenarios result in a lower overall PCC score, despite visual evidence of increased nuclear localization. Notably, the difference between DMSO-treated and DDD85646-treated samples is significant; upwards of 100 cells were measured. Further, blocking myristoylation via the G2A mutation resulted in SVIP relocating from membranes to diffuse throughout the cell (Fig. 2), including the nucleus. Nuclear localization was possibly an effect of the fluorescent tag in combination with blocking myristoylation and palmitoylation. As seen in other proteins that are dually acylated at the N-terminus, myristoylation at G2 is required for downstream palmitoylation (at C4 and C7) (Fig. 2B). When short acylated peptides are appended to fluorescent proteins, and acylation is inhibited, the proteins localize diffusely in the cytoplasm, as well as the nucleus [28–31]. Thus, the G2A mutation blocks nearly all acylation of SVIP-mCherry, leading the protein to localize like mCherry alone. Alternatively, SVIP may contain a nuclear localization signal that directs non-acylated SVIP to the nucleus.

Interestingly, the C4S substitution has similar effects on SVIP-mCherry localization as the G2A mutation (Fig. 2). This is likely due to the decreased myristoylation detected by click chemistry in the C4S mutation (Fig. 2B). Substitution of C4 to alanine did not restore myristoylation (data not shown). As previously mentioned, SVIP does not have a canonical myristoylation consensus motif, which may make it more sensitive to substitutions at the N-terminus. As expected, blocking palmitoylation at C7 did not alter myristoylation (Fig. 2A), while it visually had an intermediate effect on localization, the change was significant (Fig. 2C).

Prior evidence suggests that SVIP directs VCP localization to the plasma membrane and lysosomes [17]. Here we confirm that VCP-GFP localization is dependent on SVIP-mCherry acylation (Fig. 3A). Together, the two proteins appear to induce the formation of autophagosomes in an SVIP acylation-dependent mechanism. Again, there is an intermediate effect when only palmitoylation at C7 is blocked (C7S), which leads to SVIP-mCherry and VCP-GFP becoming concentrated along the edge of the nucleus rather than a general diffuse pattern as with other acylation mutants (Fig. 3A), in contrast to the partial membrane localization of C7S-SVIP-mCherry alone (Fig. 2C).

However, the reduced signal of SVIP acylation mutants upon co-immunoprecipitation of VCP suggests reduced interaction of both G2A- and C4S-SVIP-mCherry with VCP-GFP (Fig. 3B). The interaction between SVIP and VCP may likely rely on SVIP localization and, thus, acylation status. SVIP is the only VCP co-factor known to assemble with each VCP subunit of the VCP hexamer (6 SVIP:6 VCP monomers) [12, 18]. Therefore, immunoprecipitation of any SVIP is likely to co-immunoprecipitate VCP, but SVIP co-immunoprecipitation with VCP may not be detected due to competition with many other co-factors.

In an effort to confirm our results with VCP mutants linked to MSP, we found that R155H-VCP-GFP has a rapid cytotoxic interaction with WT-SVIP-mCherry, wherein expression of both proteins results in cell death soon after expression (Supplementary Videos 7 and 11). This detrimental interaction is prevented when SVIP-mCherry acylation is blocked via site mutations or biochemical inhibitors, suggesting that SVIP acylation impacts its interaction with the disease-causing mutant of VCP. There is an intermediate attenuation of cell death with C7S and 2-BP treatment, indicating blocking myristoylation may be required for complete prevention of cytotoxicity. Specifically, there may be reduced interaction of R155H-VCP with G2A- and C4S-SVIP, resulting in rescue of protein expression and cell death. As mentioned previously, there is some indication in the co-immunoprecipitation results that the acylation mutants of SVIP may have reduced interaction with VCP. This may carry over with R155H-VCP as well and rescue the detrimental interaction. Indeed, when the VCP-interaction motif mutant of SVIP (VIMm-SVIP) was co-expressed with R155H-VCP, it failed to co-immunoprecipitate with both wild-type and R155H-VCP (Fig. 6A). VIMm-SVIP also rescued the adverse phenotype when co-expressed with R155H-VCP (Fig. 6). This further supports the hypothesis that the interaction between R155H-VCP and SVIP may be modulated through SVIP myristoylation, and, perhaps, through SVIP palmitoylation at C4.

The adverse phenotype observed between R155H-VCP-GFP and WT-SVIP-mCherry in our studies contrasts previous findings [16]. In *D. melanogaster*, SVIP knockout results in significant muscular, mitochondrial, and neuronal degeneration while restoring muscular SVIP expression rescues many of these degenerative effects, including neuronal degeneration [16]. This may be explained by a combination of factors. First, our system uses overexpression of mCherry-tagged SVIP and GFP-tagged VCP. However, the protein and cell death were confined to the disease variant R155H and not observed with WT-VCP-GFP. Cell death was also observed when SVIP-mCherry was co-expressed with R155H-VCP-GFP in multiple cell types (HEK293T, HeLa, and primary hippocampal neurons—not shown). Finally, protein and cell death were particular to SVIP-mCherry acylation status, which likely differs in *D. melanogaster*. In flies, SVIP has an additional cysteine at position 8 as well as a more canonical myristoylation site (M**G**A**C**LS**CC**GQ…), in comparison to mammalian SVIP, which only has cysteines at positions 4 and 7, and a non-canonical myristoylation site (M**G**L**C**FP**C**PGE…). Specifically, F5, P6, and P8 in human SVIP are not ideal amino acids for myristoylation. This suggests that human SVIP is not fully myristoylated, and therefore palmitoylated, and may exist in multiple forms based on its acylation status (i.e., there may be pools of acylated and non-acylated SVIP). Thus, *D. melanogaster* SVIP is likely a better substrate for myristoylation and more efficiently palmitoylated, due to the third cysteine, than mammalian SVIP, affecting its localization and function differently than in mammals. Additionally, while VCP is highly conserved from yeast to humans [32], SVIP does not appear to be as well conserved between species (35% sequence identity between fly and human SVIP in contrast to ~85% for VCP [33]). This difference in conservation may be reflective of diverse functions in different species. It may suggest that as humans developed disease variants of VCP over time, SVIP acylation became less desirable. As such, it will be critical to study the role of SVIP acylation in multiple species and living systems to develop a comprehensive understanding of SVIP’s complex role in disease. That said, based on the requirement of SVIP for tubular lysosomes in *Drosophila* and those in our study, SVIP may be a membrane-curvature sensing and inducing protein that is dependent on acylation. Many membrane-curvature sensing and inducing proteins are fatty acylated [34–36]. Therefore, the potentially more efficient myristoylation and additional palmitoylation of SVIP in *Drosophila* may make SVIP a better curvature-inducing protein that facilitates the formation of tubular lysosomes.

The mechanism behind this adverse interaction needs to be explored in future experiments. One potential mechanism is alterations in autophagy. It is important to note that autophagosome-like vesicles are produced when the wild-type forms of SVIP and VCP are co-expressed in multiple tested cell types (primary hippocampal neurons (Fig. 8, column 3), HEK293T (Fig. 2A), and HeLa (not shown)). Data from previous literature indicate that these vesicles are indeed autophagosomes [17]. Since the interaction between WT-VCP and WT-SVIP results in autophagosomes and SVIP regulates VCP’s functions in autophagy, this process may become aberrantly regulated with R155H-VCP and WT-SVIP, rapidly deteriorating the cells expressing both proteins. Other possible mechanisms of protein and cellular toxicity include ERAD and apoptosis, both of which VCP is typically involved in [4]. However, the resistance to caspase inhibitors suggests a caspase-independent form of cell death.

The regulation of VCP and SVIP interaction by acylation will be a critical avenue to explore. The results demonstrate the importance of both SVIP myristoylation and palmitoylation in modulating its regulation of VCP localization and the resulting protein and cellular toxicity with the most common disease-causing variant of VCP. These results warrant further investigations toward the mechanism of this interaction. Knowing the multitude of roles played by VCP, investigating the contributions of ER stress (e.g., PERK, grp78/BIP) and other markers of caspase-dependent pathways (PARP-1, BAX, CHOP) would aid in explaining the mechanism of protein and cellular toxicity. However, PARP and caspase-3 cleavage were not detected, nor did grp78/BIP increase in our systems (data not shown), suggesting a potentially unconventional form of cell death. Further, VCP is present in the cell’s nucleus and cytoplasm and is associated with membranes [9]. As such, investigating cellular fractions to determine VCP and SVIP localization would suggest the probable location and pathway for adverse interaction. These investigations are key next steps and will require developing a system in which the resultant cell death occurs less rapidly. Developing cell lines in which WT-SVIP is knocked out, or R155H-VCP is knocked in constitutively [37] would allow the measurement of markers of different pathways more efficiently. Determining whether the adverse interaction between WT-SVIP and R155H-VCP can be rescued by knocking out endogenous SVIP is important. While the mCherry and R155H-VCP conditions indicate that lack of WT-SVIP prevents the adverse interaction, knocking out endogenous SVIP before R155H-VCP expression would suggest a therapeutic avenue that may be independent of acylation.

While SVIP acylation is a novel target in MSP, currently available small molecules would target global acylation with potential off-target effects [38]. However, our groups and others have successfully developed drug screens to identify new inhibitors of S-acylation [39]. Others have generated peptides to break toxic interactions between ZDHHCs and their substrates [40–42]. As the ZDHHCs that acylate SVIP are uncovered, novel approaches for targeting SVIP acylation may arise. Alternatively, targeting SVIP expression through antisense oligonucleotides, siRNA, or other genetic targeting approaches may also prove effective. Nevertheless, studying SVIP acylation in MSP is critical to understanding the disease and development of therapeutics.

## Methods

### Cell culture and transfection

HEK293T cells (ATCC; CRL 11268), pre-tested for mycoplasma (https://www.atcc.org/products/crl-11268) were grown at 37 °C in 5% CO_2_ in Dulbecco’s Modified Eagle Medium (DMEM; Wisent # 319-005-CL, supplemented with 10% FBS, 0.1% penicillin–streptomycin, 0.1% l-glutamine, 0.1% sodium pyruvate). Cells were seeded at 300,000 cells/well for biochemical experiments or 200,000–225,000 cells/well in 6-well dishes for microscopy experiments. The next day, cells were transfected with 5 µg of plasmid DNA using calcium phosphate precipitation and incubated for 2 h, as previously described [39]. Cells were processed 18 h post transfection.

### Mouse-derived primary hippocampal neurons

Primary hippocampal neurons were derived from mouse (FVBN/J strain) embryos at embryonic day 17 (E17). Embryonic hippocampi were dissociated using papain and seeded at 180,000 cells/well for microscopy experiments on 0.1 mg/mL poly-l-lysine coated wells or glass coverslips (#1.5, VWR) in 6-well plates. They were plated in plating media (neurobasal supplemented with 2.5% FBS, 2.5% horse serum, pen–strep, l-Glutamine, and B27). The next day, the media was changed to neuronal media (neurobasal supplemented with pen–strep, l-glutamine, and B27). At 14 days in vitro, cells were transfected with lipofectamine 2000 (Invitrogen) and fixed the following day using 4% paraformaldehyde in 1× phosphate-buffered saline (PBS).

### Cloning

SVIP is predicted to be myristoylated at Glycine 2 by the prediction programs TermiNator (https://bioweb.i2bc.paris-saclay.fr/terminator3/test.php) and SVMyr (https://busca.biocomp.unibo.it/lipipred/3a05fb48-b5c8-40f5-93e0-71427220d8bf/showresult/). SVIP is predicted to be palmitoylated at Cysteines 4 and 7 by CSS-Palm 3.0 (https://gpspalm.biocuckoo.cn/download.php [22]). All SVIP-mCherry plasmids were generated using gBlock or gene strands (IDT or Eurofins, respectively). Briefly, the open reading frame of SVIP was transferred from the plasmid into FEW-mCherry viral vector (kind gift from Dr. Gareth Thomas, Temple University) using the XhoI and NotI restrictions enzyme sites, resulting in a C-terminal mCherry tag. The resulting constructs are referred to as WT-SVIP-mCherry in the following text. G2A, C4S, C7S, C4,7S, and G2A-C4,7S-SVIP-mCherry refer to the SVIP site mutants in the FEW-mCherry vector. Additionally, a gBlock was used to introduce three point mutations (R22E, A26V, R32E) in the VIM region in WT-SVIP-mCherry (VIMm-SVIP-mCherry) (see Fig. 1A for a summary of the site mutations).

The open reading frames of WT-VCP-eGFPN1, R155H-VCP-eGFPN1, and A232E-VCP-eGFPN1 [11] were transferred from the eGFP plasmid into FEW-GFP viral vector using standard molecular cloning techniques with the NotI and SalI restriction enzyme sites, resulting in a C-terminal GFP tag. The FEW-GFP vector is typically cut with NotI and XhoI; however, since VCP contains a XhoI cut site, SalI was used instead. The resulting plasmids were used for all experiments and are referred to as WT-VCP-GFP and R155H-VCP or R155H-VCP-GFP in the following text.

### Fatty acid labeling and click chemistry

Cell labeling and click chemistry were performed as described previously [43, 44]. Briefly, cells were deprived of lipids for 45 min–1 h in DMEM supplemented with delipidated FBS (Life Technologies). Then, 15 min prior to labeling, cells were treated with the indicated inhibitors [1 µM DDD85646 dissolved in DMSO or 20 µM 2-BP dissolved in ethanol] where indicated. Next, alkyne-tagged fatty acid analogs (alkyne-myristate (13-tetradecylnoic acid; 13-TDYA; Click Chemistry Tools 1164) or alkyne-palmitate (15-hexadecynoic acid; 15-HDYA; Click Chemistry Tools 1165)) were saponifed in potassium hydroxide. Cells were then incubated with the saponified alkyne fatty acid analogs for 3 h, after which cells were lysed in modified RIPA buffer (50 mM HEPES pH 7.4, 150 mM NaCl, 0.5% sodium deoxycholate, 2 mM MgCl_2_, 0.1% SDS, 1% Igepal CA-630). Rabbit anti-mCherry (Cell Signaling) and goat anti-GFP (Eusera) were used to immunoprecipitate (IP) SVIP-mCherry and VCP-GFP, respectively. Immunoprecipitates (IPs) and lysates (input) were subjected to click chemistry with tris-carboxyethylphosphine (TCEP), copper sulfate, *S*-(benzyltriazolylmethyl)amine (TBTA), and azide-647. All reagents for Click Chemistry were purchased from Click Chemistry Tools (Vector Labs).

### Acyl-biotin exchange (ABE)

The ABE was performed as previously described [39, 45]. Briefly, cells were sonicated and lysed in lysis buffer (50 mM HEPES, 2% SDS, 1 mM EDTA) with 50 mM *N*-ethylmaleimide (NEM). Lysates were incubated at 50 °C for 2 h to block free thiols with NEM. NEM was then removed during a 2 h incubation at room temperature, rotating in the presence of 2,3-dimethyl-1,2-butadiene, added to a final concentration of 4%, followed by chloroform precipitation of butadiene (final concentration of 8%) and phase separation of excess NEM. The top phase was collected and incubated with 0.7 M hydroxylamine and 4 mM HPDP-Biotin (Soltec) for 1 h rotating at room temperature to cleave thioester bonds. After an overnight 80% acetone precipitation at −20 °C, protein pellets were washed with 80% acetone, and resuspended in lysis buffer without NEM. Following protein quantification, 500 µg–1 mg of protein was incubated in 150 mM NaCl dilution buffer (50 mM HEPES, 1% TX-100, 1 mM EDTA, 1 mM EGTA) with High-Capacity Neutravidin Agarose beads (Thermo #29202) at 4 °C for 4 h. Following two washes with 0.5 M NaCl dilution buffer and one wash with dilution buffer without NaCl, the protein was eluted from beads with elution buffer (0.2% SDS, 250 mM NaCl, 1% *β*-mercaptoethanol) over a 10 min incubation at 37 °C, then denatured at 95 °C for 5 min in 5X sample-loading buffer with 5% *β*-mercaptoethanol, and stored at −20 °C.

### Western blotting

10% Bis–Tris acrylamide gels were run in XT MOPS buffer (Biorad #1610788) and transferred onto nitrocellulose using the Transblot Turbo (Biorad). Rat anti-mCherry (1:1000, Invitrogen), and rabbit anti-GFP (1:10,000, Eusera) were used to detect SVIP-mCherry and VCP-GFP, respectively. Fluorescent secondaries anti-mouse Starbright 700 (Biorad) and anti-rabbit Starbright 520 (Biorad) were used. Tubulin-rhodamine (Biorad) was used to detect tubulin as the loading control.

### Fluorescence microscopy

For fixed cell imaging, HEK293T cells were seeded at 200,000–225,000 cells/well onto 0.01 mg/mL poly-d-lysine coated coverslips (#1.5, VWR) in 6-well plates. After an 18 h transfection (described above), cells were washed once with 1× PBS, then fixed in 4% paraformaldehyde for 20 min at room temperature. After two PBS washes, cells were incubated in 1 µg/mL DAPI (Sigma D9542) in PBS for 20 min at room temperature, followed by three PBS washes. The coverslips were mounted onto microscope slides (VWR 48311-601) using ProLong Gold antifade mountant (Invitrogen P36934), and sealed with nail polish. Images were acquired using the Nikon AXR laser scanning confocal microscope at 20× magnification. Using Nikon AXR software, z-stack images at 20× were taken, which were first denoised, followed by deconvolution, and the resulting maximum intensity projections are reported below.

Hippocampal neurons seeded at 180,000 cells/well onto 0.1 mg/mL poly-l-lysine coated coverslips (#1.5, VWR) in 6-well plates. After an 18 h transfection, cells were fixed in 4% paraformaldehyde for 20 min as described above. Images were acquired using the Nikon AXR laser scanning confocal microscope at 60× magnification. Z-stacks images were taken, which were denoised, followed by deconvolution, and the resulting maximum intensity projections are reported below.

For live-cell imaging, HEK293T cells were plated at ~225,000 cells/well on poly-d-lysine coated 1.5 coverglass in 35 mm live-cell culture dishes (MatTek). The next day, cells were transfected with the indicated constructs. After ~2 h, media was replaced and cells were incubated with LysoTracker Deep Red. In order to capture dying cells, cells expressing R155H-VCP-GFP and WT-SVIP-mCherry were imaged overnight at 60× magnification and maintained at 37 °C at 5% CO_2_. All other conditions were imaged the next day under the same conditions.

### Quantification

Five images were randomly selected by the Nikon AXR Program for Figs. 1–6 with the exception of Fig. 3, where three images per condition were taken by the user. Z-stacks were obtained at 20×, and 60× for Fig. 3. Images were denoised and deconvolved. Nuclear intensity, cell volume, DAPI-protein PCC, and total VCP cell counts were taken using programs generated in Nikon’s General Analysis 3 (GA3). For nuclear protein intensity, a threshold around DAPI and mCherry (SVIP) was made, DAPI that had mCherry signal was isolated, and sum or mean intensity values for each object were taken. For cell volume, a threshold around mCherry or overlapping mCherry and GFP (VCP) signal(s) was made and the total volume per field of view was measured. For DAPI-protein PCC, DAPI and mCherry signals were thresholded separately, DAPI having mCherry signal was isolated, and PCC for each object was taken. For total cell counts, a threshold around the DAPI signal was made, and the number of objects was counted. For VCP cell counts, a threshold around DAPI and GFP was made, DAPI having GFP signal was isolated, and the number of objects was counted. VCP-SVIP PCC for Fig. 3 did not use a program generated in GA3, instead max-intensity projections of the images were made, regions of interest were manually drawn around single cells, and PCC was taken.

### Statistical analyses

All quantitative data were analyzed using GraphPad Prism 10. Data were analyzed by ANOVAs, with a significant *p* value of 0.05. Significant ANOVA results were followed up by post-hoc analyses using Tukey’s test to identify the differences between groups. Sample sizes and biological repeats are indicated in the figure legends.

### Supplementary information

Supplement Video Descriptions Ramzan et al

Uncropped Gels

Supplemental Video 1 [green channel] Live cell imaging of HEK293T cells transfected with WT-VCP-GFP and WT-SVIP-mCherry

Supplemental Video 2 [red channel] Live cell imaging of HEK293T cells transfected with WT-VCP-GFP and WT-SVIP-mCherry

Supplemental Video 3 [merge_green_red] Live cell imaging of HEK293T cells transfected with WT-VCP-GFP and WT-SVIP-mCherry

Supplemental Video 4 [green channel] Live cell imaging of HEK293T cells transfected with WT-VCP-GFP and G2A-SVIP-mCherry

Supplemental Video 5 [red channel] Live cell imaging of HEK293T cells transfected with WT-VCP-GFP and G2A-SVIP-mCherry

Supplemental Video 6 [merge_green_red] Live cell imaging of HEK293T cells transfected with WT-VCP-GFP and G2A-SVIP-mCherry

Supplemental Video 7 [merge] Live cell imaging of HEK293T cells transfected with R155H-VCP-GFP, WT-SVIP-mCherry, and Cathepsin B-BFP

Supplemental Video 8 Live cell imaging of HEK293T cells transfected with WT-VCP-GFP, WT-SVIP-mCherry, and LysoTracker-DeepRed

Supplemental Video 9 Live cell imaging of HEK293T cells transfected WT-VCP-GFP, G2A-SVIP-mCherry, and LysoTracker-DeepRed

Supplemental Video 10 Live cell imaging of HEK293T cells transfected with R155H-VCP-GFP, G2A-SVIP-mCherry, and LysoTracker-DeepRed

Supplemental Video 11 Live cell imaging of HEK293T cells transfected with R155H-VCP-GFP, WT-SVIP-mCherry, and LysoTracker-DeepRed

## Data Availability

Data have not been uploaded to a repository and are available upon request.
