# Supplementary material for: Fatty links between multisystem proteinopathy and small VCP-interacting protein
Source: Cell Death Discov. 2024 Aug 8;10:358. doi: 10.1038/s41420-024-02118-9 (PMC11310202; doi:10.1038/s41420-024-02118-9)
Supplement: Supplementary file 1 — Supplement Video Descriptions Ramzan et al [file 41420_2024_2118_MOESM1_ESM.docx]

**Supplemental Video 1 [green channel] Live cell imaging of HEK293T cells transfected with WT-VCP-GFP and WT-SVIP-mCherry**

Description: WT-VCP-GFP (green) is co-expressed with WT-SVIP-mCherry in HEK293T cells. This video demonstrates live VCP localization in the cell 18 hours post-transfection.

**Supplemental Video 2 [red channel] Live cell imaging of HEK293T cells transfected with WT-VCP-GFP and WT-SVIP-mCherry**

Description: WT-VCP-GFP is co-expressed with WT-SVIP-mCherry (red) in HEK293T cells. This video demonstrates live SVIP localization in the cell 18 hours post-transfection.

**Supplemental Video 3 [merge_green_red] Live cell imaging of HEK293T cells transfected with WT-VCP-GFP and WT-SVIP-mCherry**

Description: WT-VCP-GFP (green) is co-expressed with WT-SVIP-mCherry (red) in HEK293T cells. This video shows the overlap between the green and red channels, showing the overlap between VCP and SVIP localization. Localization of SVIP and VCP at membranes of lysosome-like structures is evident.

**Supplemental Video 4 [green channel] Live cell imaging of HEK293T cells transfected with WT-VCP-GFP and G2A-SVIP-mCherry**

Description: WT-VCP-GFP (green) is co-expressed with G2A-SVIP-mCherry in HEK293T cells. This video demonstrates live VCP localization in the cell 18 hours post-transfection.

**Supplemental Video 5 [red channel] Live cell imaging of HEK293T cells transfected with WT-VCP-GFP and G2A-SVIP-mCherry**

Description: WT-VCP-GFP is co-expressed with G2A-SVIP-mCherry (red) in HEK293T cells. This video demonstrates live SVIP localization in the cell 18 hours post-transfection.

**Supplemental Video 6 [merge_green_red] Live cell imaging of HEK293T cells transfected with WT-VCP-GFP and G2A-SVIP-mCherry**

Description: WT-VCP-GFP (green) is co-expressed with G2A-SVIP-mCherry (red) in HEK293T cells. This video demonstrates the overlap between live VCP and SVIP localization in the cell 18 hours post-transfection. There is a lack of localization of VCP and SVIP at the vesicle membranes.

**Supplemental Video 7 Live cell imaging of HEK293T cells transfected with R155H-VCP-GFP, WT-SVIP-mCherry, and Cathepsin B-BFP**

Description: R155H-VCP-GFP (green) was co-expressed with WT-SVIP-mCherry (red) and Cathepsin B-BFP (magenta) in HEK293T cells. This video demonstrates live localization of R155H-VCP, WT-SVIP, and cathepsin B as the cell expresses the proteins and dies.

**Supplemental Video 8 Live cell imaging of HEK293T cells transfected with WT-VCP-GFP, WT-SVIP-mCherry, and LysoTracker-DeepRed**

Description: Live cell time-lapse of HEK cells transfected with WT-VCP-GFP (green) and WT-SVIP-mCherry (red). LysoTracker-DeepRed (cyan) was added 1hr prior to imaging.

**Supplemental Video 9 Live cell imaging of HEK293T cells transfected WT-VCP-GFP, G2A-SVIP-mCherry, and LysoTracker-DeepRed**

Description: Live cell imaging of HEK cells transfected with WT-VCP-GFP (green) and G2A-SVIP-mCherry (red). LysoTracker-DeepRed (cyan) was added 1hr prior to imaging.

**Supplemental Video 10 Live cell imaging of HEK293T cells transfected with R155H-VCP-GFP, WT-SVIP-mCherry, and LysoTracker-DeepRed**

Description: Live cell imaging of HEK cells transfected with R155H-VCP-GFP (green) and WT-SVIP-mCherry (red). LysoTracker-DeepRed (cyan) was added 1hr prior to imaging.

**Supplemental Video 11** **Live cell imaging of HEK293T cells transfected with R155H-VCP-GFP, G2A-SVIP-mCherry, and LysoTracker-DeepRed**

Description: Live cell time-lapse of HEK cells transfected with R155H-VCP-GFP (green) and G2A-SVIP-mCherry (red). LysoTracker-DeepRed (cyan) was added 1hr prior to imaging.
