# Supplementary material for: Fatty links between multisystem proteinopathy and small VCP-interacting protein
Source: Cell Death Discov. 2024 Aug 8;10:358. doi: 10.1038/s41420-024-02118-9 (PMC11310202; doi:10.1038/s41420-024-02118-9)

FIGURE 1B

SVIP +/- DDD or 2BP alk-myr/alk-palm Click 10% Bis-Tri Gels, NT, Clicked with AFdye647

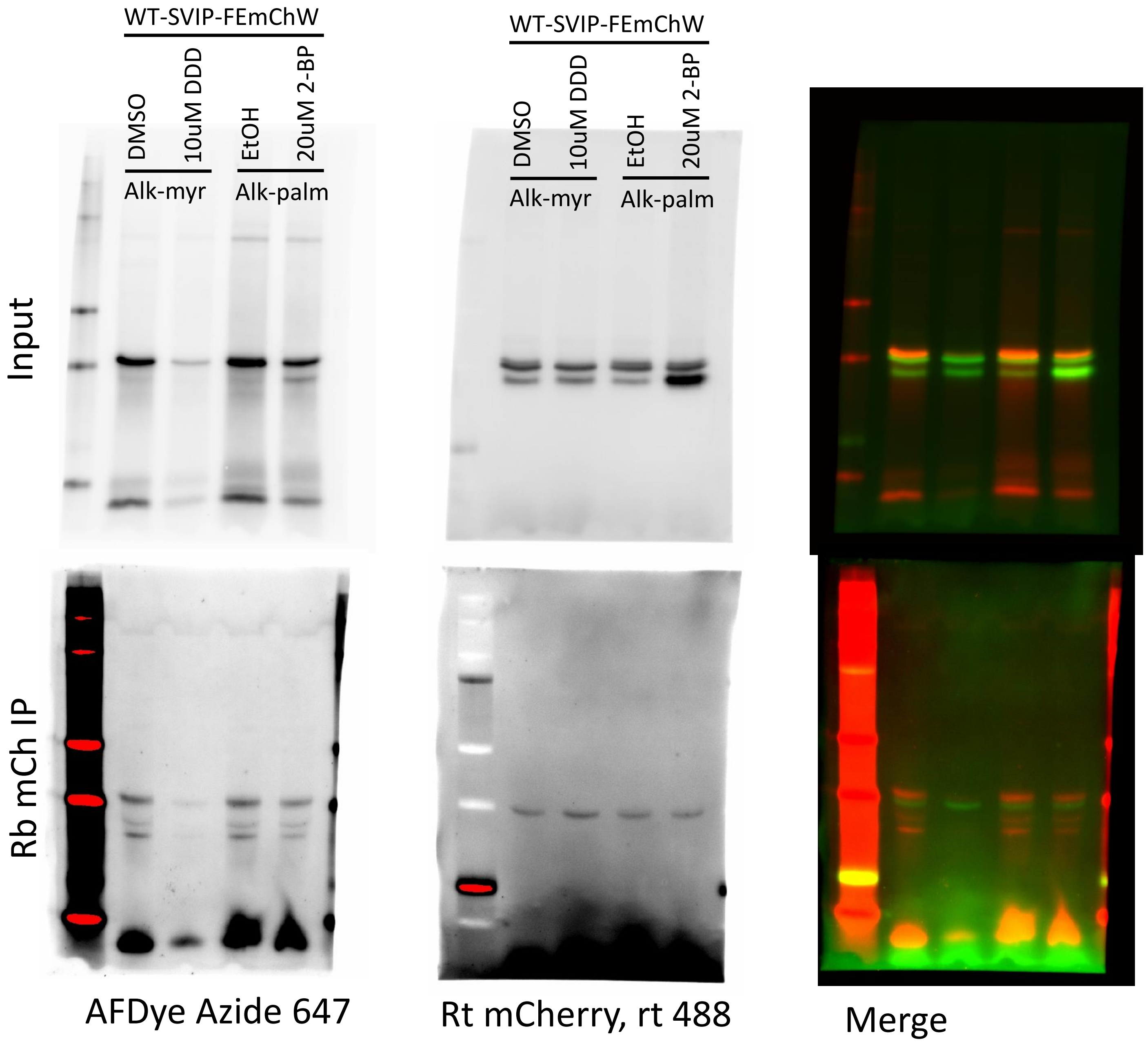

FIGURE 2A

SVIP Site-Mutants alk-myr Click

10% Bis-Tri Gels, NT, Clicked with AFDye Azide 647

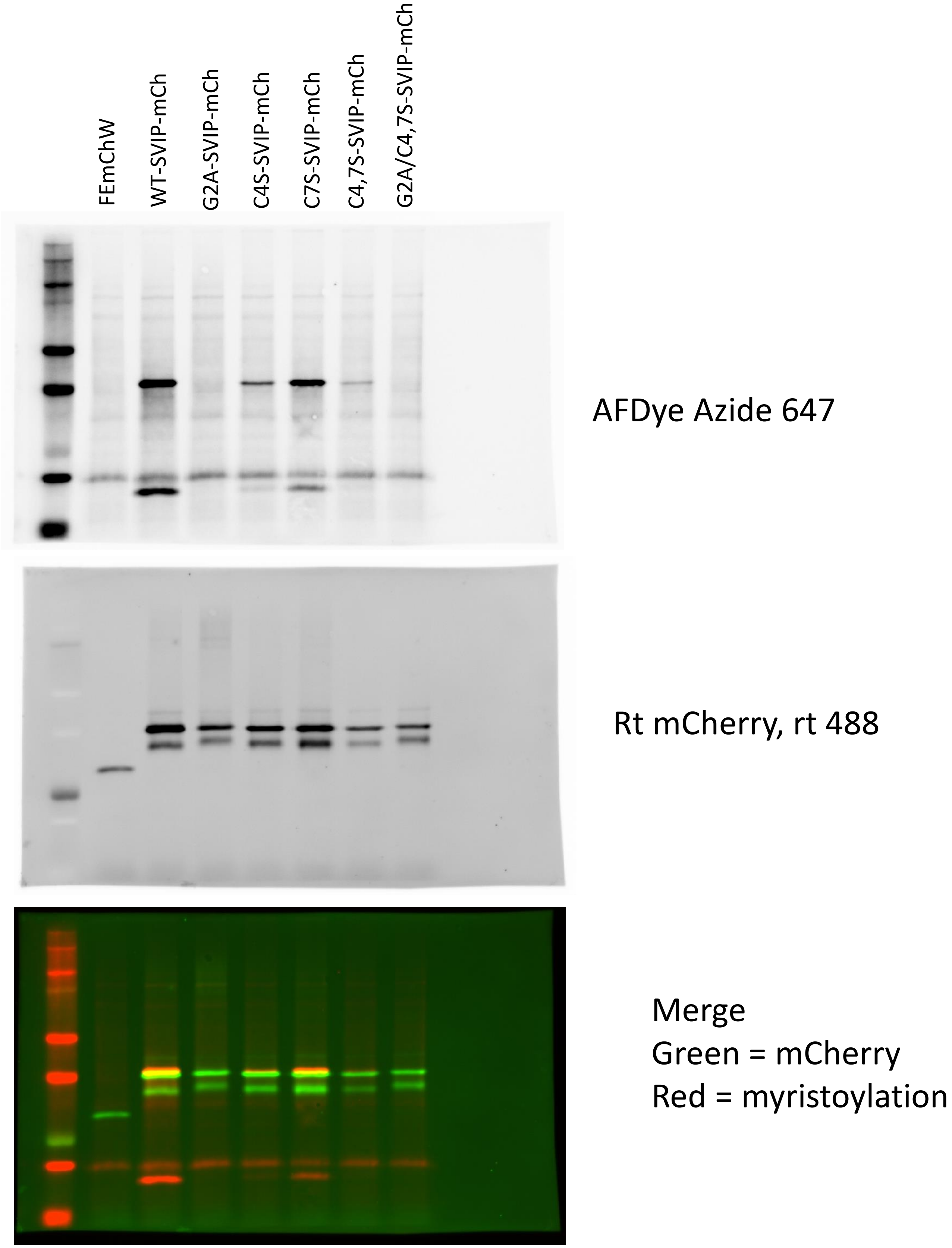

FIGURE 2B – WT/mutant SVIP ABE

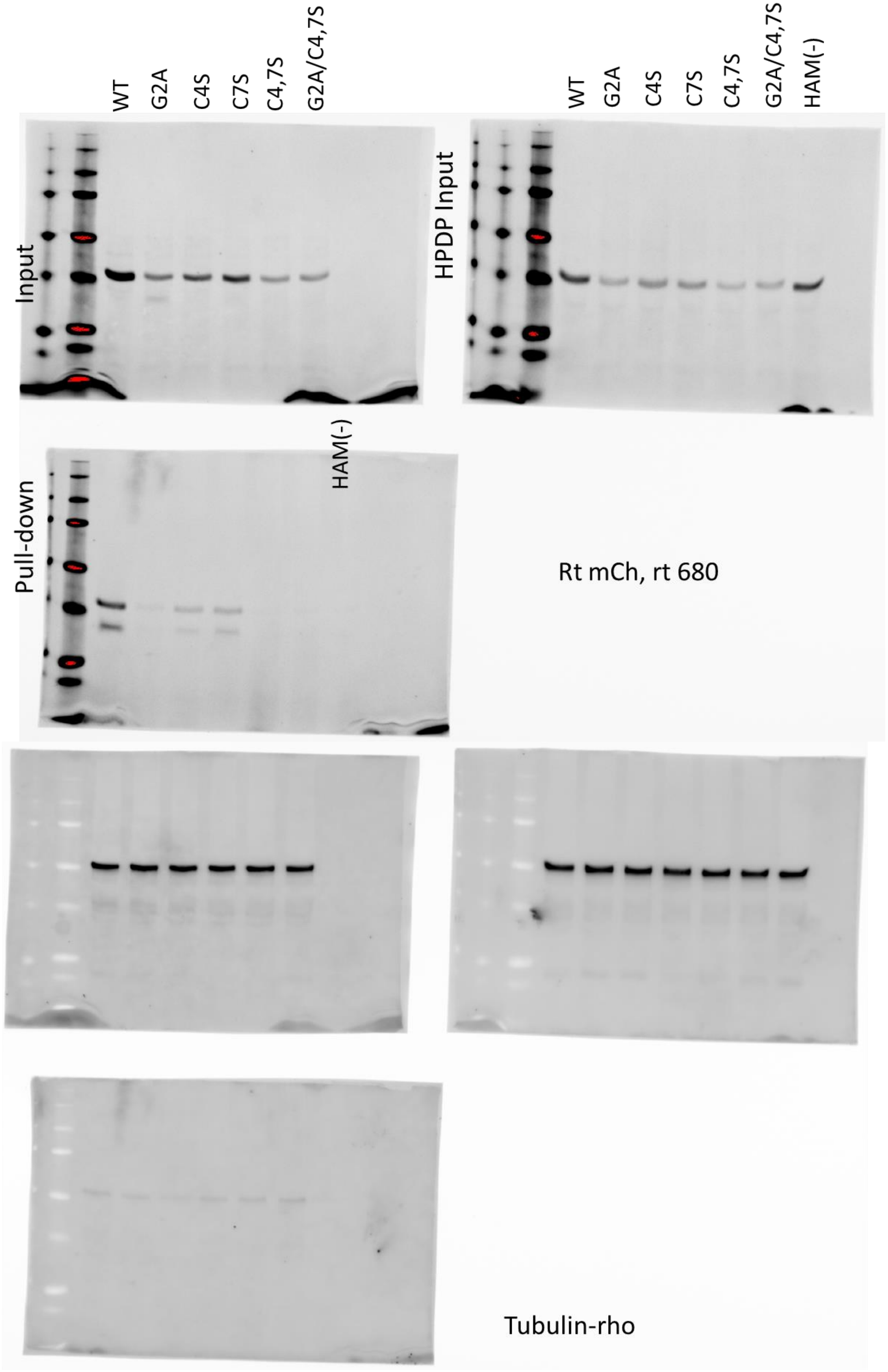

FIGURE 3B – rabbit mCherry IP, wt VCP + wt/mt SVIP

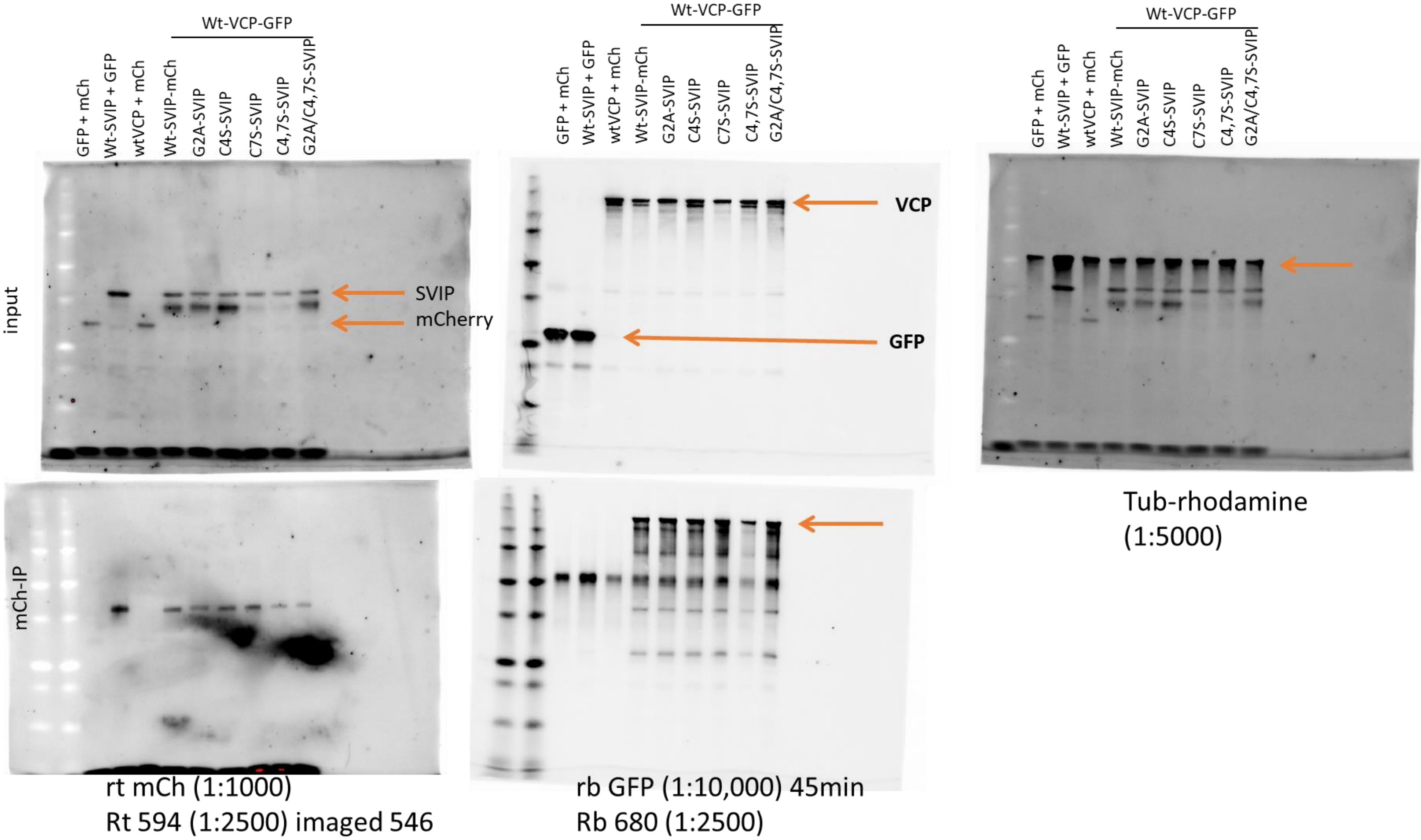

FIGURE 3B – goat GFP-IP, wt VCP + wt/mt SVIP

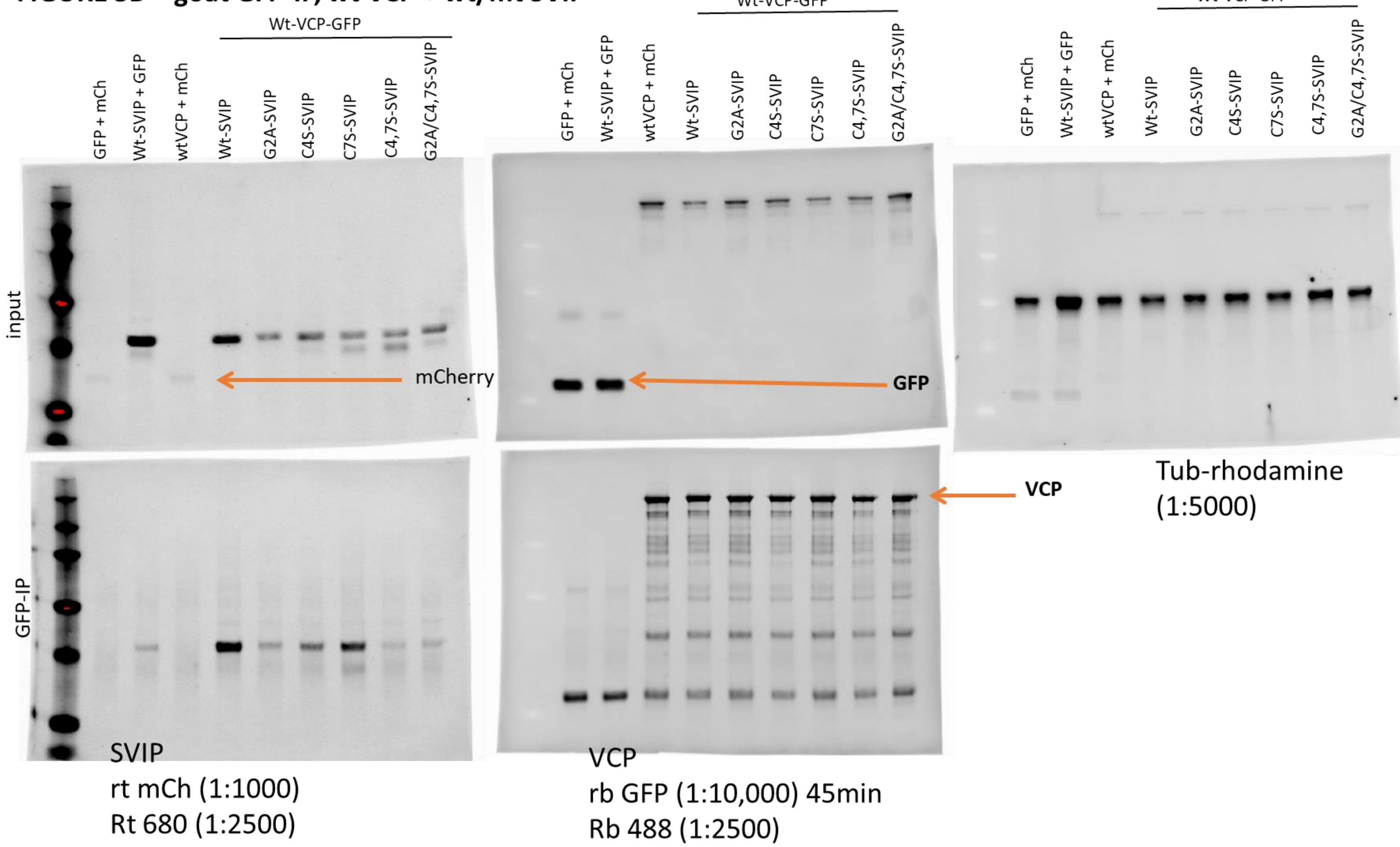

FIGURE 4A: goat GFP-IP, wt/mt SVIP + wt/mt VCP

mCh = mCherry

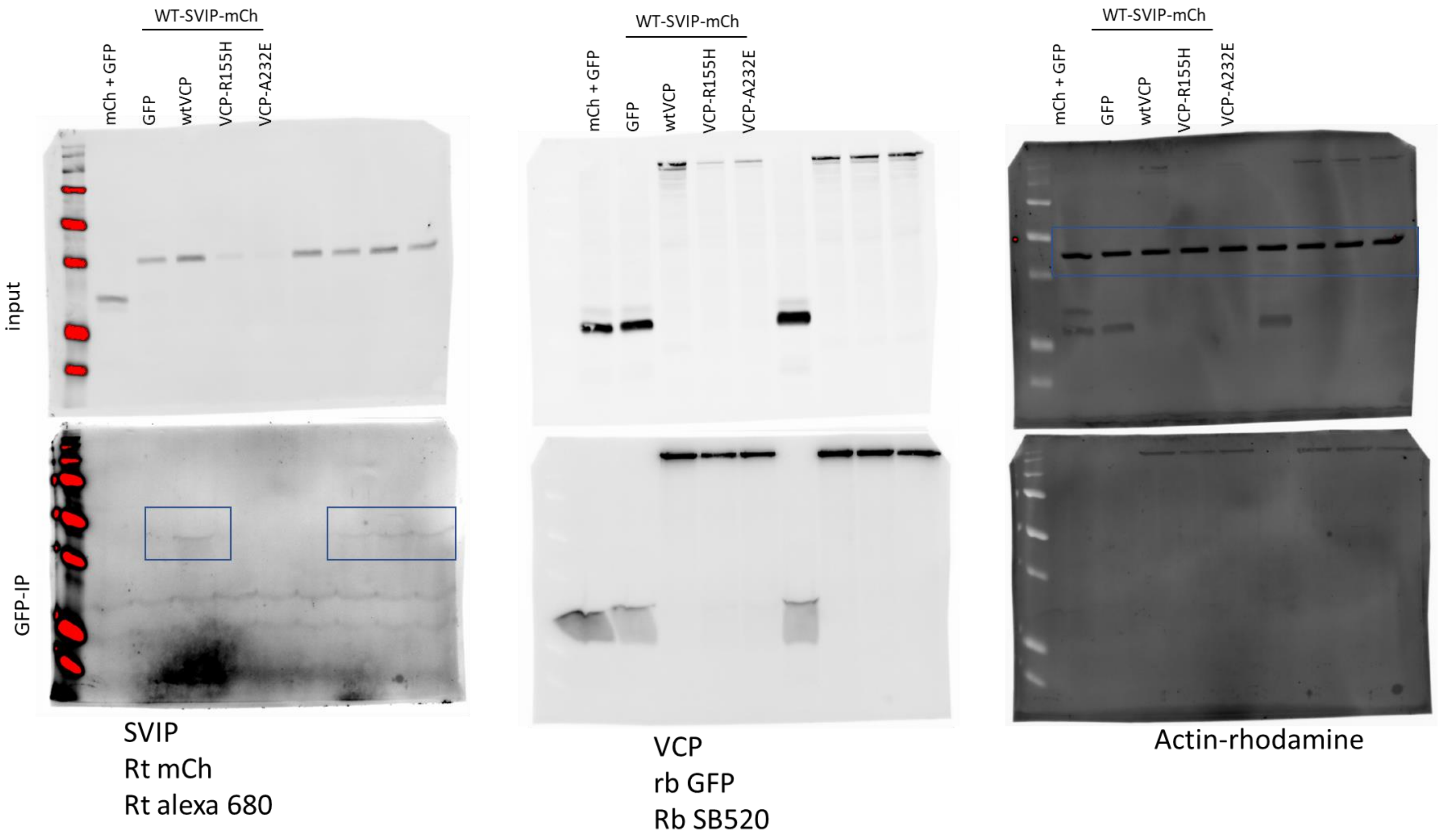

FIGURE 4A: rabbit mCherry IP, wt/mt SVIP + wt/mt VCP

mCh = mCherry

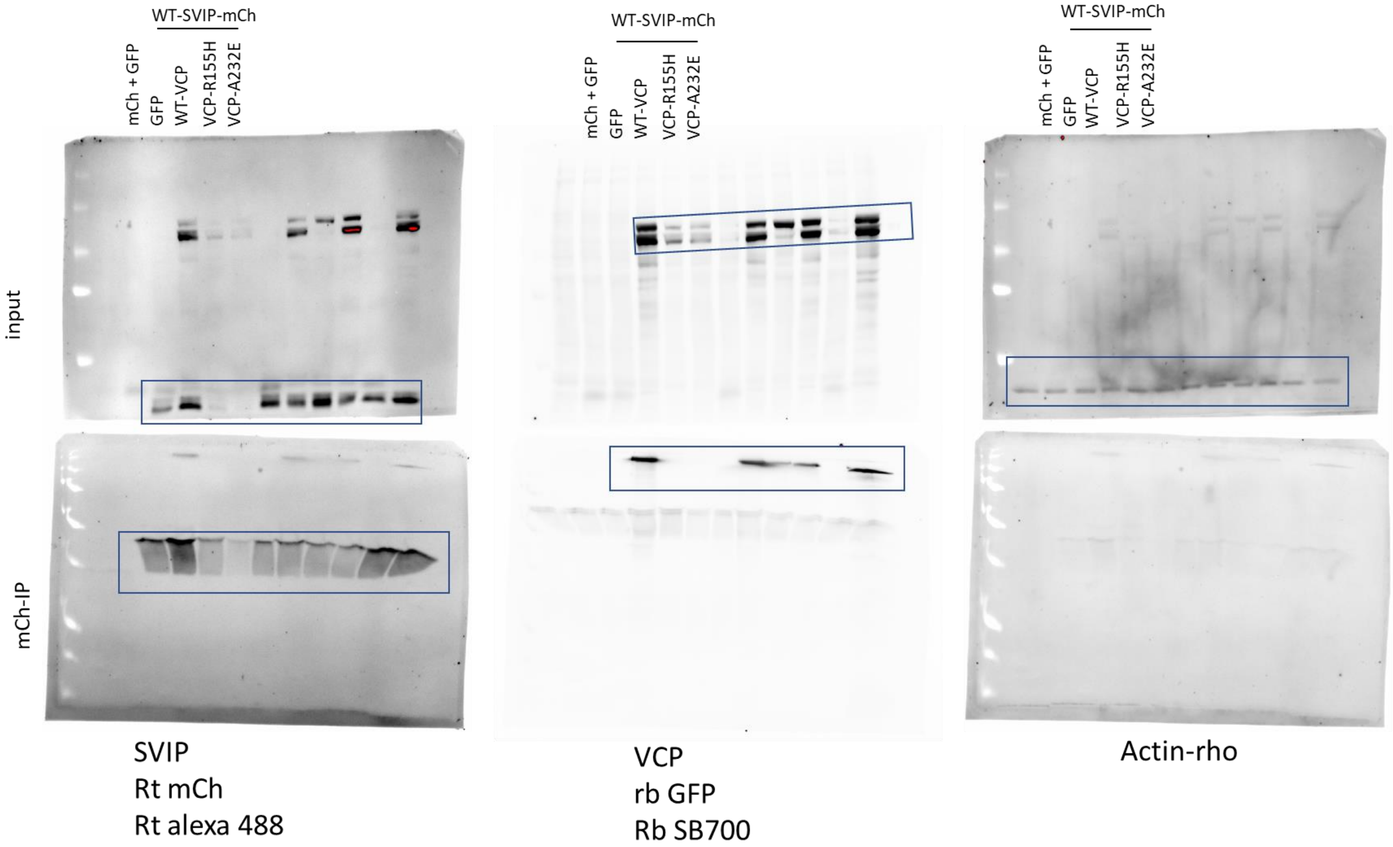

FIGURE 4B

20230315-17 SVIP + WT/R155H-VCP + 10uM ZVAD in DMSO (18hr treatment) + lysed w ZVAD in buffer

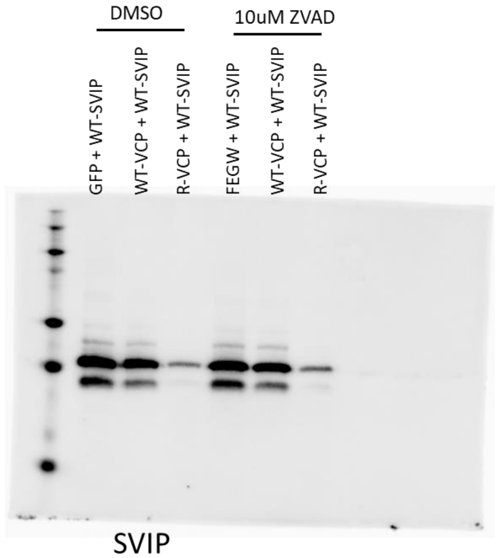

SVIP
Rt mch (1:1000) ON
Rt 680 (1:2500) 1hr

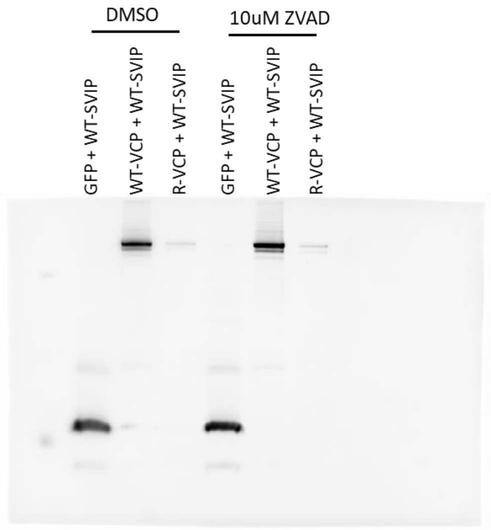

VCP
Rb GFP(1:10,000) 1hr
Rb 488 (1:2500) 1hr

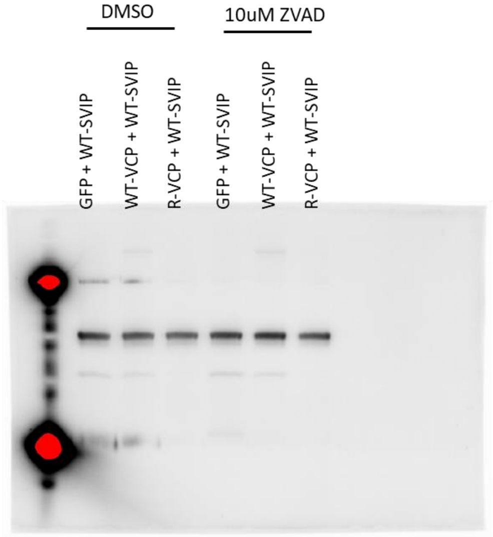

Tubulin-rho (1:5000) 1hr

FIGURE 4C: R155H-VCP + palm SVIP mutants

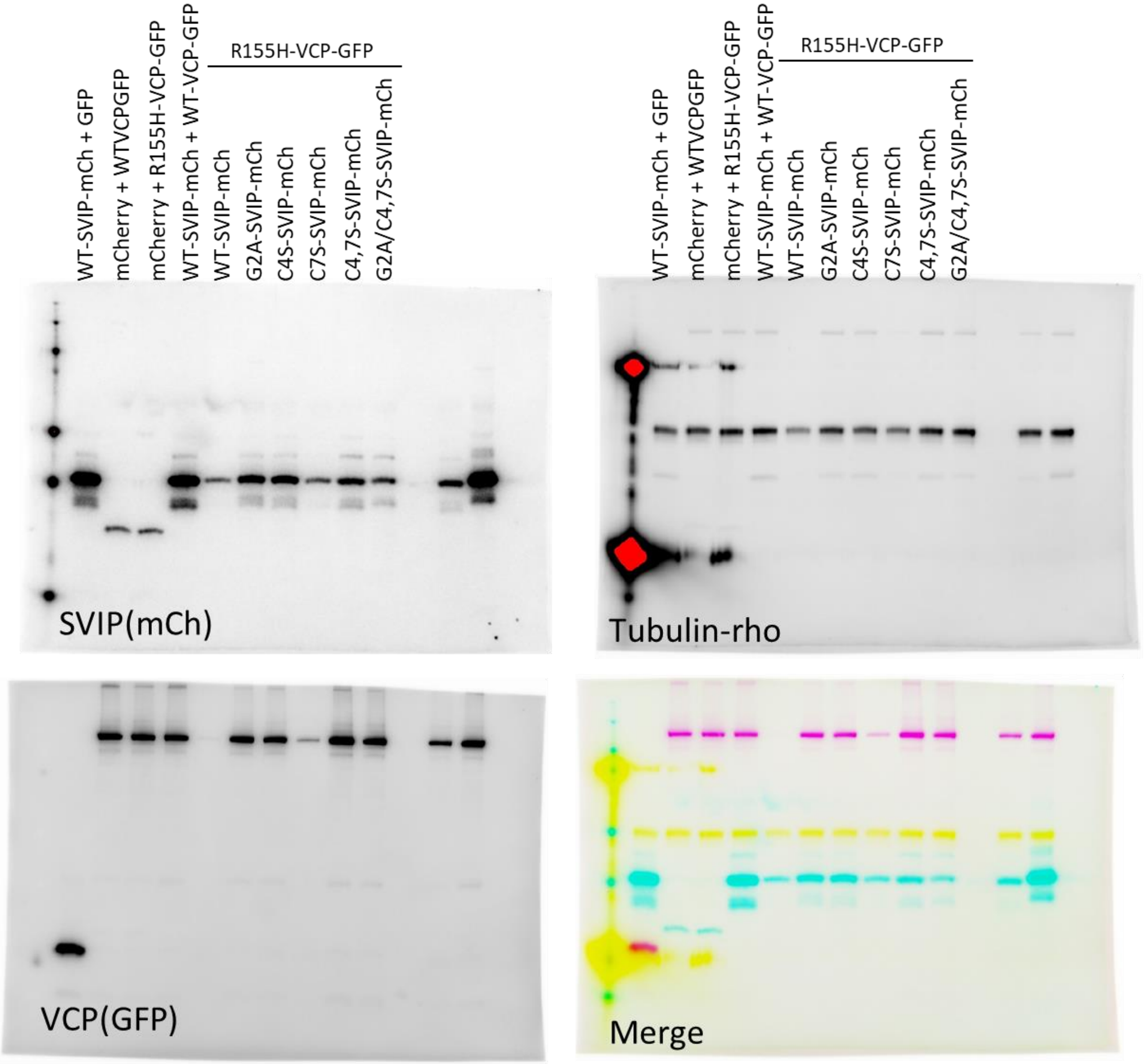

1. rt mCh (1:1000) O/N + rt 680 (1:2500)
2. Rb GFP (1:10,000) 1hr + rb 488 (1:2500)
3. Tub-rhodamine (1:5000) 1hr

FIGURE 5B: R-VCP + wtSVIP DDD 2BP rescue

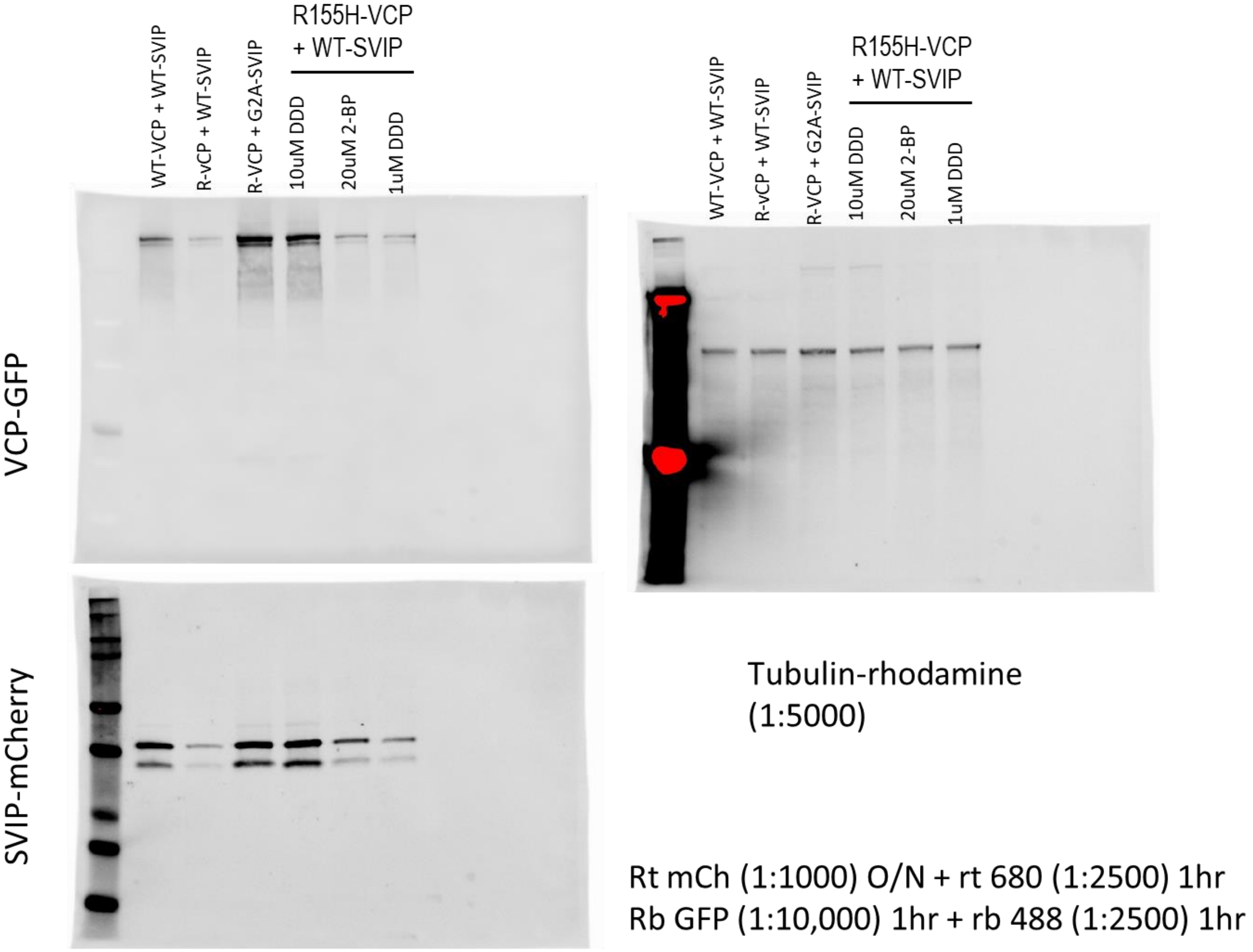

FIGURE 6A: mCh-IP (rb-anti-mCh)

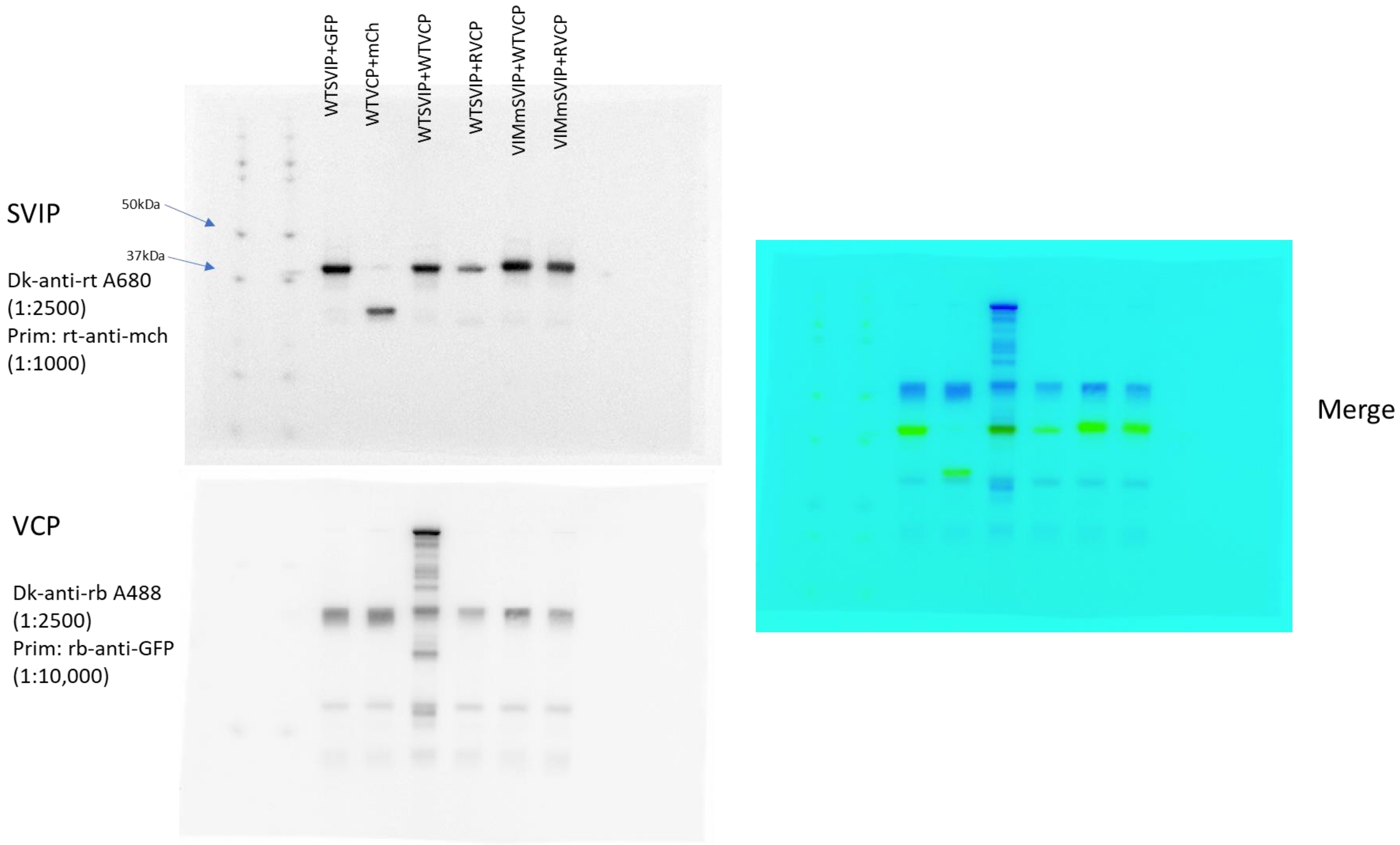

FIGURE 6A: GFP-IP (gt-anti-GFP)

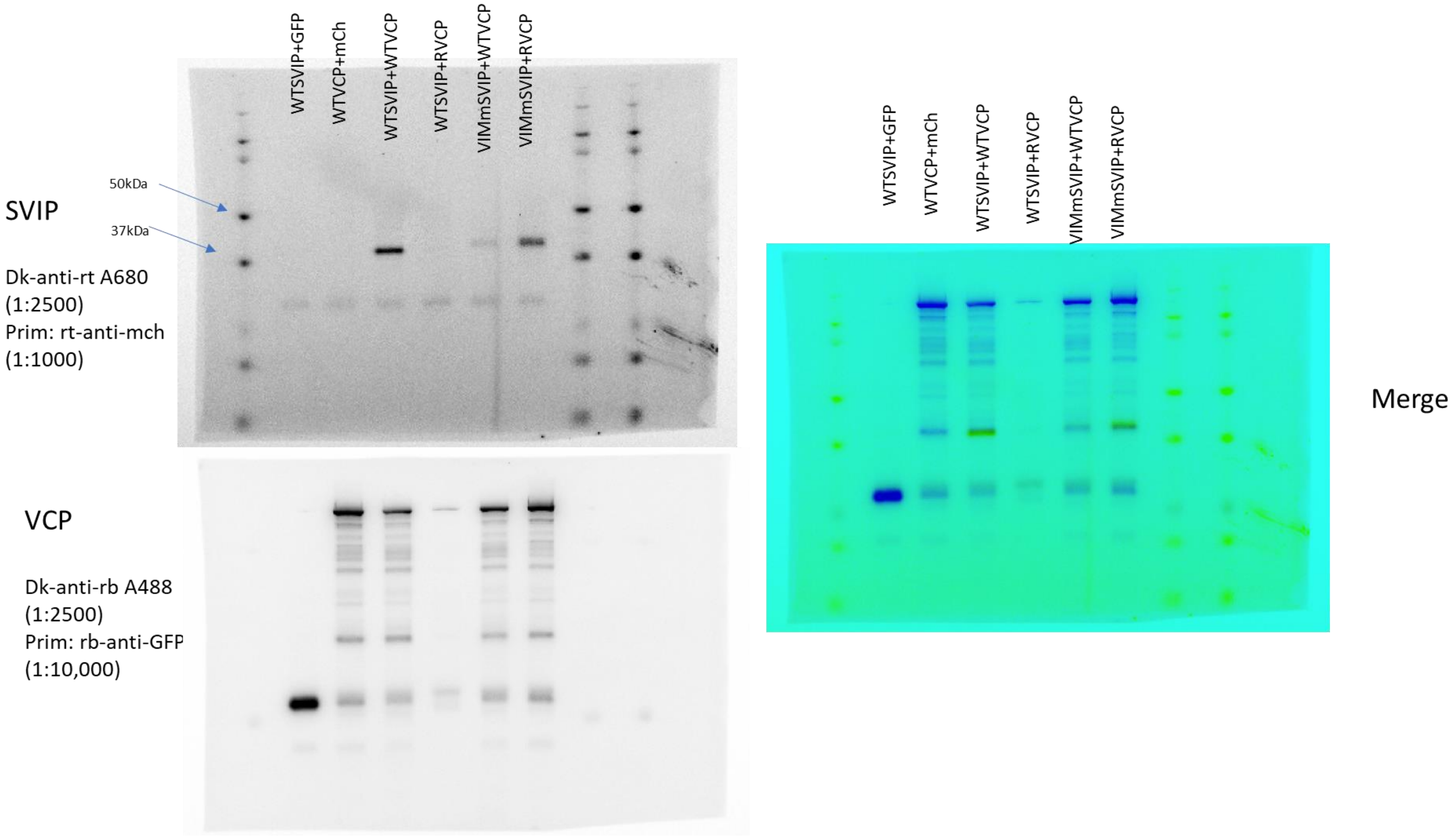

FIGURE 6A: Inputs

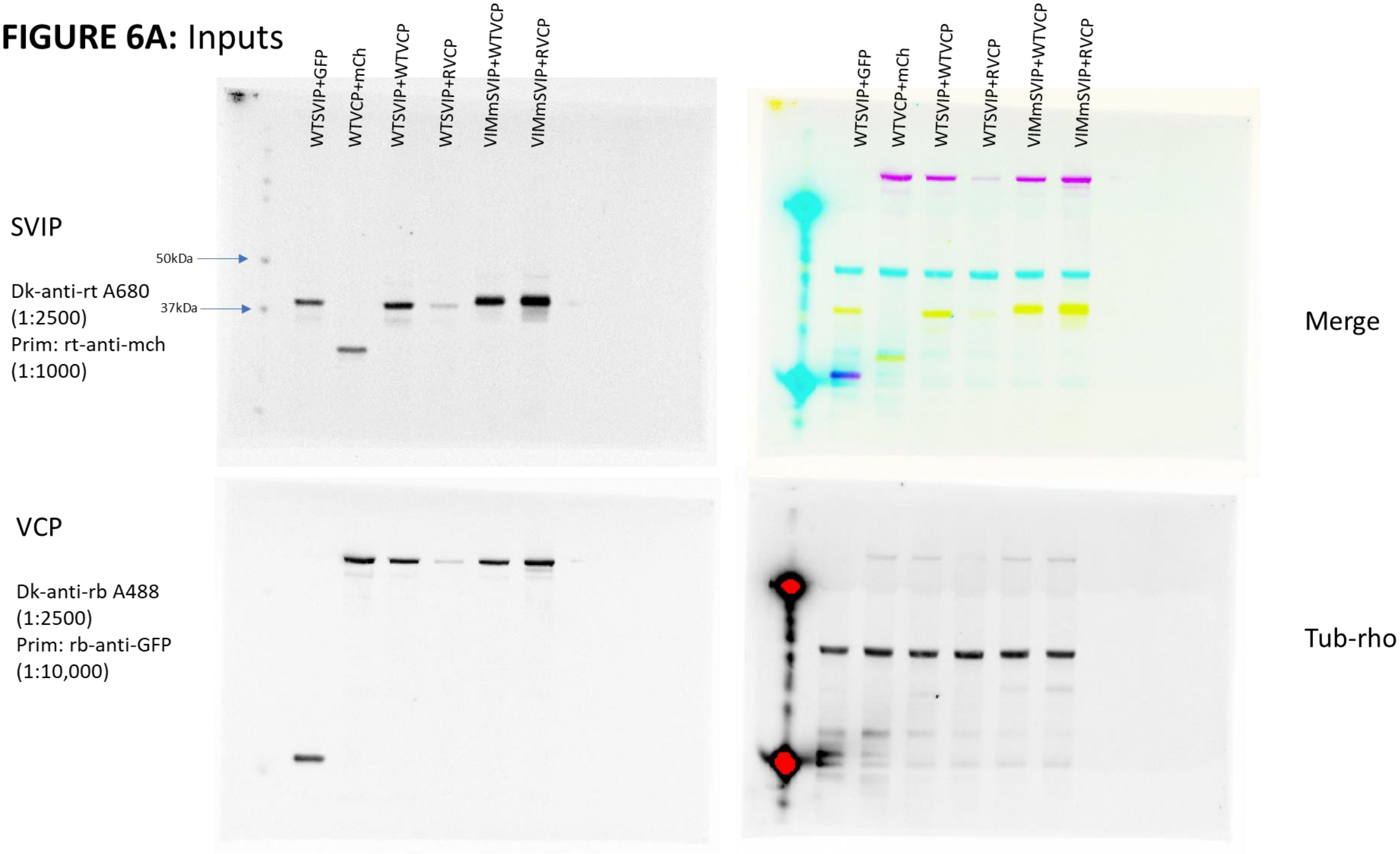

Supplement: Supplementary file 2 — Uncropped Gels [file 41420_2024_2118_MOESM2_ESM.pdf]
